# A PGE_2_-MEF2A axis enables context-dependent control of inflammatory gene expression

**DOI:** 10.1016/j.immuni.2021.05.016

**Published:** 2021-08-10

**Authors:** Francesco Cilenti, Giulia Barbiera, Nicoletta Caronni, Dario Iodice, Elisa Montaldo, Simona Barresi, Eleonora Lusito, Vincenzo Cuzzola, Francesco Maria Vittoria, Luca Mezzanzanica, Paolo Miotto, Pietro Di Lucia, Dejan Lazarevic, Daniela Maria Cirillo, Matteo Iannacone, Marco Genua, Renato Ostuni

**Affiliations:** 1 Vita-Salute San Raffaele University, Milan, Italy; 2 San Raffaele Telethon Institute for Gene Therapy (SR-Tiget), Milan, Italy; 3 Genomics of the Innate Immune System Unit, IRCCS San Raffaele Scientific Institute, Milan, Italy; 4 Emerging Bacterial Pathogens Unit, Division of Immunology, Transplantation and Infectious Diseases, IRCCS San Raffaele Scientific Institute, Milan, Italy; 5 Dynamics of Immune Responses Unit, Division of Immunology, Transplantation and Infectious Diseases, IRCCS San Raffaele Scientific Institute, Milan, Italy; 6 Center for Omics Sciences (COSR), IRCCS San Raffaele Scientific Institute, Milan, Italy; 7 Experimental Imaging Centre, IRCCS San Raffaele Scientific Institute, Milan, Italy

**Keywords:** macrophages, inflammation, innate immunity, transcription, chromatin, cytokines, LPS, interferons, MEF2, PGE2

## Abstract

Tight control of inflammatory gene expression by antagonistic environmental cues is key to ensure immune protection while preventing tissue damage. Prostaglandin E_2_ (PGE_2_) modulates macrophage activation during homeostasis and disease, but the underlying mechanisms remain incompletely characterized. Here we dissected the genomic properties of lipopolysaccharide (LPS)-induced genes whose expression is antagonized by PGE_2_. The latter molecule targeted a set of inflammatory gene enhancers that, already in unstimulated macrophages, displayed poorly permissive chromatin organization and were marked by the transcription factor myocyte enhancer factor 2A (MEF2A). Deletion of MEF2A phenocopied PGE_2_ treatment and abolished type I interferon (IFN I) induction upon exposure to innate immune stimuli. Mechanistically, PGE_2_ interfered with LPS-mediated activation of ERK5, a known transcriptional partner of MEF2. This study highlights principles of plasticity and adaptation in cells exposed to a complex environment and uncovers a transcriptional circuit for IFN I induction with relevance for infectious diseases or cancer.

## Highlights

• PGE_2_ suppresses IFN I induction by activated macrophages
• PGE_2_ targets a set of poorly permissive inflammatory gene enhancers marked by MEF2A
• Loss of MEF2A causes functional inactivation of inflammatory gene enhancers
• MEF2A is required for IFN I induction in response to multiple innate immune stimuli

## Graphical abstract

**Figure:**
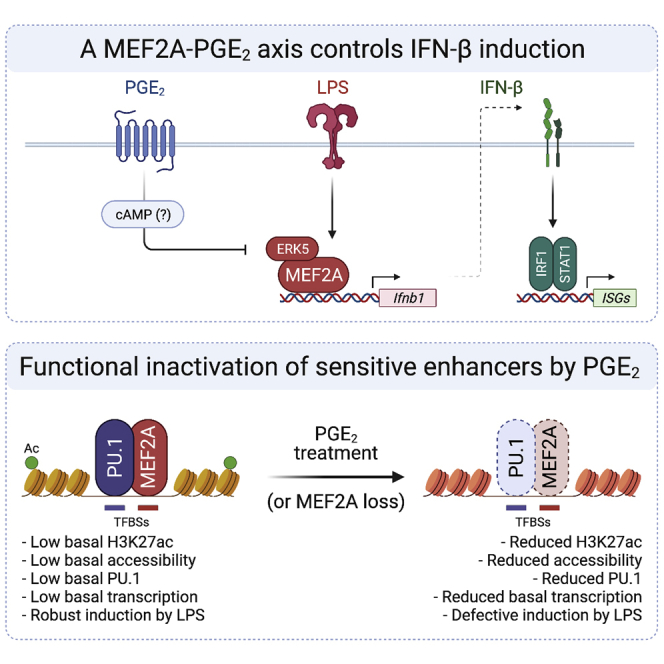

## Teaser

Prostaglandin E_2_ (PGE_2_) modulates macrophage activation during homeostasis and disease, but the underlying mechanisms remain incompletely characterized. Cilenti, Barbiera, et al. reveal that PGE_2_ suppresses inflammatory gene expression by targeting a set of inflammatory gene enhancers marked by MEF2A, a critical regulator of type I interferon induction.

## Introduction

Dynamic changes in gene expression enable biological processes during development and homeostasis and in response to stress. Innate immune cells, such as macrophages, are paradigmatic examples of how transcriptional plasticity drives complex functional outputs (Pope and Medzhitov, 2018; Smale, 2010). Upon exposure to pathogenic insults, macrophages coordinately express hundreds of genes encoding for antimicrobial effectors, inflammatory cytokines, as well as positive and negative feedback regulators (Bhatt et al., 2012; Xue et al., 2014). This occurrence reflects locus-specific chromatin remodeling at promoters and enhancers, driven by combinations of lineage-determining and stimulus-activated transcription factors (TFs) (Garber et al., 2012; Ghisletti et al., 2010; Heinz et al., 2010; Kaikkonen et al., 2013; Ostuni et al., 2013).

Inflammatory gene expression must be tightly regulated to prevent tissue damage. For instance, type I interferons (IFN I) protect against infection or cancer by stimulating cytotoxic cells (Demaria et al., 2019) but may cause immune-mediated pathology (Barrat et al., 2019). Macrophages use cell-intrinsic and -extrinsic control systems to ensure proper expression of transcripts encoding for potentially toxic products. On one hand, promoters of the latter genes tend to be poorly permissive so that transcriptional induction requires chromatin remodeling (Bhatt et al., 2012; Hargreaves et al., 2009; Nicodeme et al., 2010; Ramirez-Carrozzi et al., 2006; Saccani et al., 2001). On the other hand, inflammatory gene expression is controlled by immune-modulatory signals that co-exist in the same local milieu (Natoli and Ostuni, 2019).

The arachidonic acid derivative prostaglandin E_2_ (PGE_2_) orchestrates tissue immune homeostasis by acting on stem, epithelial, and stromal cells (Ho et al., 2017; North et al., 2007; Roulis et al., 2020). PGE_2_ has complex immune-regulatory properties that include cell recruitment and vasodilation, elicitation of regulatory programs in leukocytes, and inhibition of cytotoxic responses (Hangai et al., 2016; Luan et al., 2015; Perkins et al., 2018; Rodríguez-Ubreva et al., 2017; Sanin et al., 2018). PGE_2_ is a driver of immune modulation in the tumor microenvironment (Caronni et al., 2021), and blockade of this molecule is a target of combinatorial immunotherapies (Böttcher et al., 2018; Hou et al., 2016; Veglia et al., 2019; Zelenay et al., 2015). Along the same line, key immune modulators, such as interleukin-10 (IL-10) or IL-4, are essential for tissue homeostasis but may also dampen anti-tumor immunity (Ip et al., 2017; Maier et al., 2020; Minutti et al., 2017; Saraiva et al., 2020).

Despite the physiological relevance of these processes, the molecular mechanisms through which PGE_2_ or other immune-modulatory agents interfere with inflammatory gene expression remain incompletely characterized. We addressed this issue by performing a systematic genomic and functional analysis in macrophages exposed simultaneously to activators and modulators of innate immune responses. Our study highlights principles underlying selective control of inducible transcription by antagonistic signals and identifies determinants of PGE_2_-driven immune modulation in macrophages.

## Results

### PGE_2_, IL-10, and IL-4 target distinct sets of LPS-inducible genes

**Figure 1:**
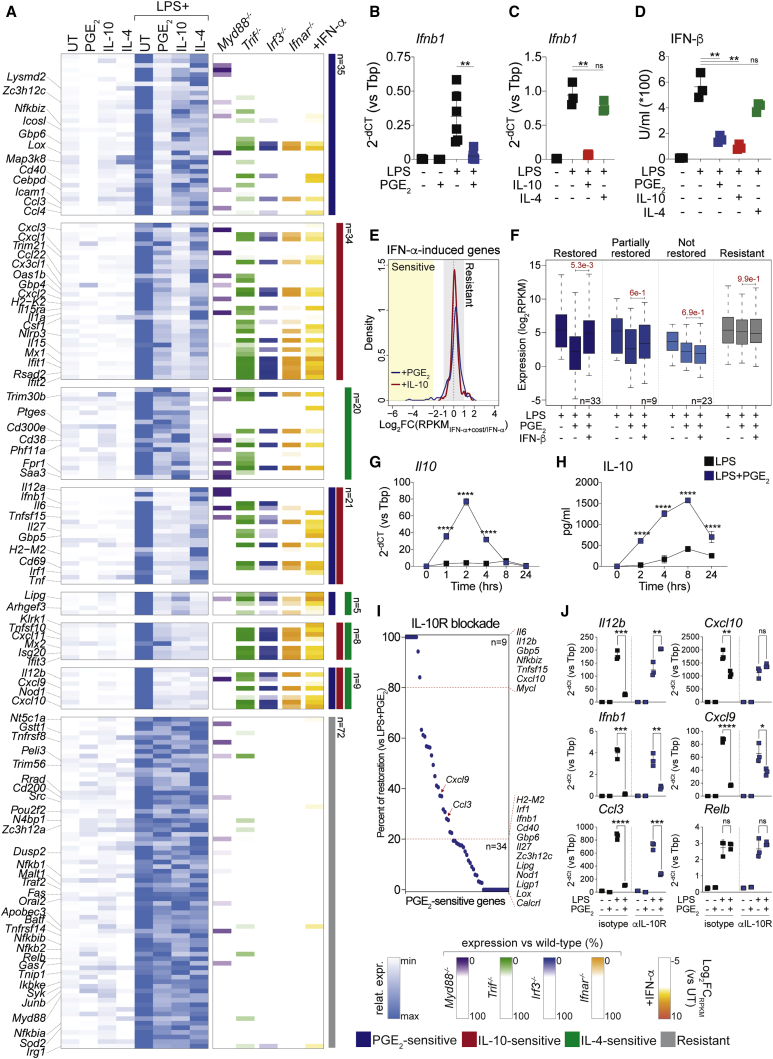
Gene-specific control of inflammatory gene expression by PGE_2_, IL-10, and IL-4 (A) Heatmap showing the behavior of LPS-inducible genes in BMDMs that are sensitive or resistant to costimulation with PGE_2_ (blue), IL-10 (red), or IL-4 (green). Left: row-normalized percentage of gene expression across experimental conditions with minimum and maximum values set to 0 and 100, respectively. Right: percentage of gene expression in lipid A-stimulated *Myd88*^*−/−*^, *Ticam1*^*−/−*^, *Irf3*^*−/−*^, and *Ifnar1*^*−/−*^ versus WT BMDMs (data from Tong et al., 2016), as well as log_2_fold change (FC) of RPKM_IFN-α_/RPKM_UT_ values. Selected gene names are shown on the left, and legends are shown at the bottom. Data are from two biological replicates. Pearson correlation > 0.97 for all replicates. (B and C) Expression of *Ifnb1* in BMDMs stimulated with LPS in the absence or presence of PGE_2_ (B), IL-10, or IL-4 (C). Dot plots represent mean ± SD. Data are from six (B) or three (C) biological replicates. ^∗∗^p < 0.01; ns, not significant (unpaired t test). (D) IFN-β release by BMDMs under the indicated conditions. The dot plot represents mean ± SD. Data are from three biological replicates. ^∗∗^p < 0.01 (unpaired t test). (E) Density plot showing the effect of costimulation with PGE_2_ or IL-10 on IFNα-induced gene expression. A dotted line indicates lack of effect of the costimulation; yellow or gray shaded areas indicate values used to define costimulation-sensitive or -resistant genes, respectively. (F) Mean expression values of resistant genes (gray) or PGE_2_-sensitive genes, classified as IFN-β restored (dark blue), partially restored (blue), or not restored (light blue) under the indicated conditions. Numbers indicate p values for the corresponding comparisons (Mann-Whitney *U* test). Data are from three biological replicates. Pearson correlation > 0.95 for all replicates. (G) Expression of *Il10* in BMDMs stimulated with LPS in the absence or presence of PGE_2_ for the indicated time points. Dot plots represent mean ± SD. Data are from three biological replicates. ^∗∗∗∗^p < 0.0001 (two-way ANOVA). (H) IL-10 release by BMDMs stimulated as indicated. Dot plots represent mean ± SD. Data are from three biological replicates. ^∗∗∗∗^p < 0.0001 (two-way ANOVA). (I) Percentage of restoration of PGE_2_-sensitive genes in the presence of IL-10R-blocking antibody. Selected gene names are shown on the right. Data are from three biological replicates. Pearson correlation > 0.98 for all replicates. (J) qRT-PCR analysis of a set of resistant and PGE_2_-sensitive genes in BMDMs stimulated as indicated. Dot plots represent mean ± SD. Data are from three biological replicates. ^∗∗∗∗^p < 0.0001, ^∗∗∗^p < 0.001, ^∗∗^p < 0.01, ^∗^p < 0.05 (unpaired t test). See also Figure S1.

To characterize the effect of immune-modulatory signals on inflammatory gene expression, we performed RNA sequencing (RNA-seq) of mouse bone marrow-derived macrophages (BMDMs) left untreated (UT); stimulated for 4 h with lipopolysaccharide (LPS), PGE_2_, IL-10, or IL-4; or costimulated with LPS+PGE_2_, LPS+IL-10, or LPS+IL-4. We first defined LPS-inducible genes (STAR Methods) and then classified them as “PGE_2_-sensitive” (n = 70), “IL-10-sensitive” (n = 72), or “IL-4-sensitive” (n = 42) when their expression was lower in costimulated versus LPS-treated BMDMs. A stringent set of genes (n = 72) with preserved expression in costimulated cells (STAR Methods) was defined as “resistant” and used for comparison (Figures 1A, S1A, and S1B; Table S1). PGE_2_, IL-10, and IL-4 acted in a selective manner and suppressed LPS-mediated induction of genes encoding for key inflammatory cytokines (e.g., *Ifnb1*, *Il12b*, *Il6*, and *Tnf*), chemokines (*Cxcl9*, *Cxcl10*, *Ccl3*, and *Ccl4*), or TFs (*Irf1*) under one or more costimulation conditions (Figures 1A and S1B; Table S1). Overall, costimulation-sensitive genes were enriched in TRIF-, IRF3-, or IFNAR-dependent transcripts (Figures 1A and S1C; Table S1), as determined by re-analysis of published RNA-seq data (Tong et al., 2016). Sensitive genes were also induced by IFN-α (Figure 1A; Table S1), whereas resistant transcripts were less responsive to IFN I and included known regulators of the nuclear factor κB (NF-κB) or AP-1 pathways (e.g., *Nfkbia*, *Relb*, and *JunB*) (Figure 1A; Table S1).

### PGE_2_ suppresses IFN-β induction and boosts IL-10 release by costimulated macrophages

We next assessed the effect of macrophage costimulation on LPS-driven IFN I responses. PGE_2_ and IL-10, but not IL-4, suppressed IFN-β release (Figures 1A–1D) and secondary activation of STAT1, STAT2, and IRF1 (Figures S1D and S1E). PGE_2_- or IL-10-mediated inhibition of *Ifnb1* and of other sensitive genes persisted over time upon acute costimulation (Figure S1F) and was evident even when antagonistic stimuli were administered hours after LPS (Figures S1G and S1H). RNA-seq of BMDMs treated with IFN-α+PGE_2_ or IFN-α+IL-10 revealed a minor effect of the latter signals on IFN I-stimulated gene expression (Figure 1E; Table S1). These data indicate that PGE_2_ and IL-10 target IFN-β induction in activated macrophages rather than antagonizing secondary responses to IFN I. Recombinant IFN-β indeed restored expression of most PGE_2_-sensitive genes in costimulated BMDMs (Figures 1F, S1I, and S1J; Table S1). However, a fraction of sensitive genes was refractory to IFN-β reconstitution (Figures 1F, S1I, and S1J; Table S1), pointing to the existence of alternative suppressive mechanisms. We next asked whether PGE_2_ and IL-10, both of which are produced by activated macrophages, influenced each other’s actions in costimulated cells. Although PGE_2_ release was unaffected in BMDMs costimulated with LPS+IL-10 (Figure S1K), IL-10 was acutely hyperinduced at the transcript and protein levels in BMDMs treated with LPS+PGE_2_ (Figures 1G and 1H). To determine whether increased IL-10 signaling contributed to the modulatory effects of PGE_2_, we performed RNA-seq on BMDMs treated with LPS+PGE_2_ in the presence of an anti-IL-10R antibody able to neutralize IL-10-mediated antagonisms (Figure S1L). A small group of PGE_2_-sensitive genes (e.g., *Il12b*, *Il6*, and *Cxcl10*) was entirely suppressed via autocrine and/or paracrine IL-10 signaling (Figures 1I, 1J, S1I, and S1J; Table S1), whereas the majority of transcripts, including *Ifnb1*, was only partially de-repressed by IL-10R blockade (Figures 1I, 1J, S1I, and S1J; Table S1). There was a limited overlap between sensitive genes whose expression was restored by adding back IFN-β or by IL-10R blockade (Figures S1I and S1J; Table S1). We conclude that PGE_2_-mediated antagonism of LPS-induced genes reflects direct inhibition of IFN-β synthesis as well as stimulation of IL-10 release by costimulated macrophages (Figures S1M).

### PGE_2_ antagonizes LPS-induced gene expression at the single-cell level

**Figure 2:**
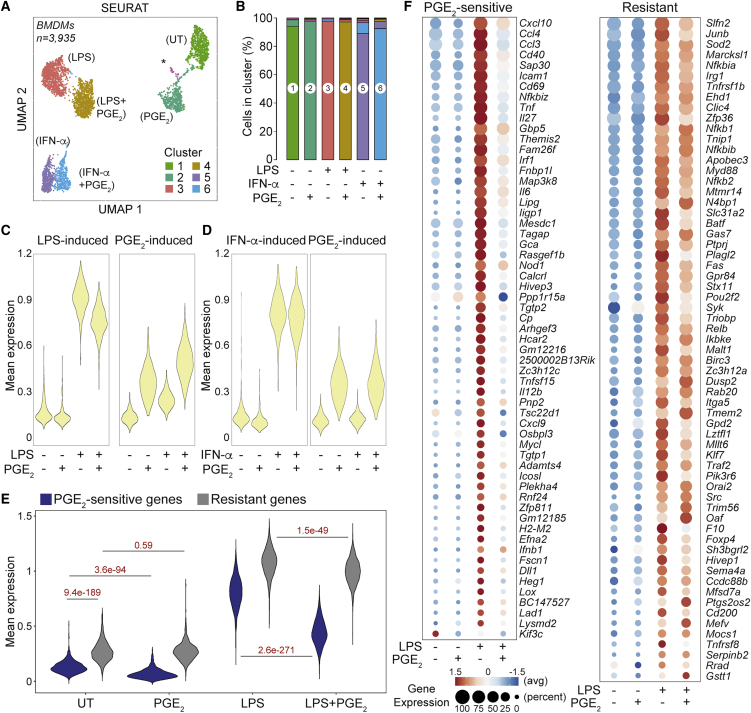
PGE_2_ antagonizes LPS-induced gene expression at the single-cell level (A) UMAP plot of scRNA-seq of BMDMs stimulated as indicated. Colors represent Louvain graph clustering, and treatments are shown in brackets. The asterisk indicates a small cluster (n = 35) of contaminant cells that were excluded from analyses. (B) Stacked bar plot showing the percentage of cells from each experimental condition within each cluster. (C and D) Mean expression values of LPS-induced (C), IFNα-induced (D), or PGE_2_-induced (C and D) genes (STAR Methods) under the indicated conditions. (E) Violin plot showing mean expression values of PGE_2_-sensitive (blue) or -resistant (gray) genes under the indicated conditions. Numbers indicate p values for the corresponding comparisons (Mann-Whitney *U* test). (F) Average expression (*Z* score) and percentage of cells expressing PGE_2_-sensitive (left) or- resistant (right) genes under the indicated conditions. Gene names are listed on the right, and legends are reported at the bottom. See also Figure S2.

We wanted to determine whether the effects of PGE_2_ on inflammatory gene expression reflected transcriptional antagonisms in individual cells or co-existence of heterogenous cellular behaviors in costimulated BMDMs. Single-cell RNA-seq (scRNA-seq) (Klein et al., 2015; Macosko et al., 2015; Zheng et al., 2017) of BMDMs left UT or stimulated for 4 h with LPS, PGE_2_, LPS+PGE_2_, IFN-α, or IFN-α+PGE_2_, followed by uniform manifold approximation and projection (UMAP) analyses (Becht et al., 2018), identified clusters corresponding to the experimental conditions tested (Figures 2A, 2B, and S2A–S2C; Table S2). In particular, most single-cell transcriptomes from BMDMs costimulated with LPS+PGE_2_ fell within the same cluster (cluster 4 in Figures 2A and S2A) and co-expressed LPS-inducible and PGE_2_-inducible genes (Figures 2C and S2C; Table S2). Virtually no costimulated BMDMs clustered with BMDMs exposed to LPS or PGE_2_ alone (Figures 2A, 2B, and S2D; Table S2), indicating that integration of antagonistic signals in single cells is the dominant behavior in our experimental setting. In keeping with bulk RNA-seq data, induction of PGE_2_-sensitive genes was reduced severely in costimulated BMDMs, whereas PGE_2_-resistant genes were less affected (Figures 2E and 2F). BMDMs costimulated with IFN-α+PGE_2_ also clustered separately from BMDMs exposed to IFN-α or PGE_2_ alone and co-expressed genes of the IFN-α- and PGE_2_-inducible programs (Figures 2A, 2D, and S2E; Table S2). However, the transcriptional difference between BMDMs exposed to IFN-α and those exposed to IFN-α+PGE_2_ was small, confirming the limited effect of PGE_2_ on IFN I-induced gene expression (Figure S2B).

### PGE_2_ antagonizes LPS-induced gene expression *ex vivo* and *in vivo*

**Figure 3:**
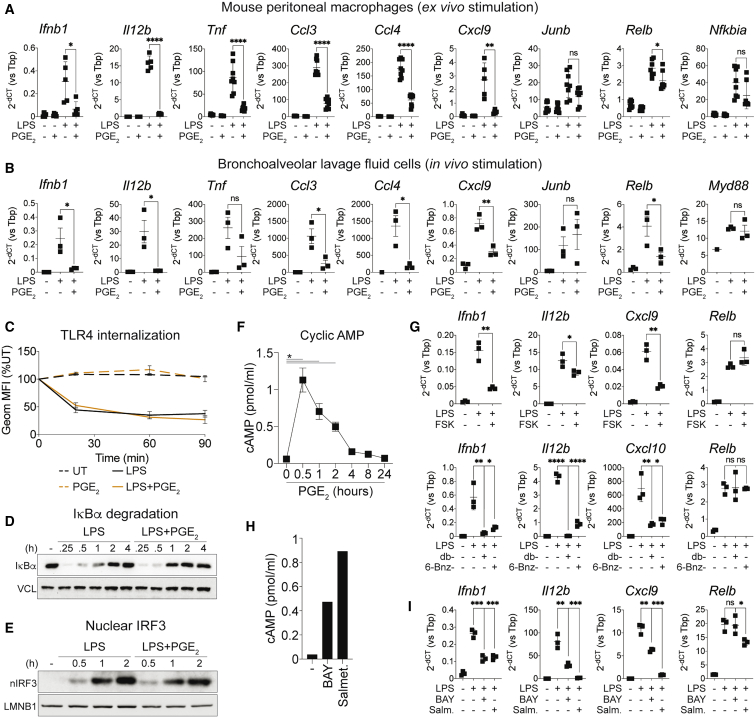
PGE_2_ antagonizes LPS-induced gene expression *ex vivo* and *in vivo* (A and B) qRT-PCR analysis of a set of PGE_2_-sensitive or -resistant genes in mouse peritoneal macrophages upon *ex vivo* stimulation (4 h) (A) or in BALF cells upon *in vivo* stimulation (2 h) (B). Dot plots represent mean ± SD. Data are from 5–8 (A) or 3 (B) biological replicates. ^∗∗∗∗^p < 0.0001, ^∗∗∗^p < 0.001, ^∗∗^p < 0.01, ^∗^p < 0.05 (unpaired t test). (C) Flow cytometry analysis of TLR4 internalization showing the percentage of geometric mean fluorescence intensity relative to the UT condition in BMDMs stimulated as indicated. The line plot represents mean ± SD. Data are from three biological replicates. (D and E) Western blot analyses of IκBα and vinculin (VCL) in whole-cell extracts (D) or of IRF3 and lamin B1 (LMNB1) in nuclear extracts (E) in BMDMs stimulated as indicated. (F) Intracellular cAMP in BMDMs treated with PGE_2_ for the indicated times. The line plot represents mean ± SD. Data are from two biological replicates. ^∗^p < 0.05 (unpaired t test). (G) qRT-PCR analysis of a set of PGE_2_-sensitive or -resistant genes in BMDMs treated for 4 h with LPS in the absence or presence of forskolin (top), dibutyryl-cAMP (db-cAMP), or 6-benzoyl-cAMP (6-Bnz-cAMP) (bottom) (2-h pre-treatment). Dot plots represent mean ± SD. Data are from three biological replicates. ^∗∗∗∗^p < 0.0001, ^∗∗∗^p < 0.001, ^∗∗^p < 0.01, ^∗^p < 0.05 (unpaired t test). (H) Intracellular cAMP in BMDMs treated for 30 min with BAY60-6583 (BAY) or salmeterol xinafoate (Salmet.). Data are from two technical replicates. (I) qRT-PCR analysis of a set of PGE_2_-sensitive or -resistant genes in BMDMs treated for 4 h with LPS in the absence or presence of BAY or salmeterol xinafoate. Dot plots represent mean ± SD. Data are from three biological replicates. ^∗∗∗^p < 0.001, ^∗∗^p < 0.01, ^∗^p < 0.05 (unpaired t test). See also Figure S3.

We next assessed whether PGE_2_-mediated immune modulation occurred in other myeloid cell populations. Defective induction of *Ifnb1* and of other sensitive genes was measured in human monocyte-derived macrophages (Figure S3A), mouse bone marrow-derived dendritic cells (Figure S3B), and mouse peritoneal macrophages (Figure 3A) treated *ex vivo* with LPS+PGE_2_. To determine whether PGE_2_ controlled inflammatory responses *in vivo*, we challenged mice with a sublethal dose of LPS, alone or in combination with PGE_2_, and assessed gene expression changes on freshly isolated peritoneal macrophages. Although no *Ifnb1* upregulation could be detected at the analyzed time point, PGE_2_-sensitive genes such as *Il12b*, *Tnf*, or *Ccl3* were induced defectively in macrophages from mice treated with LPS+PGE_2_ (Figure S3C). We next administered LPS or LPS+PGE_2_ intranasally for 2 h and isolated bronchoalveolar lavage fluid (BALF) cells, which were largely composed of lung alveolar macrophages (Figure S3D). In keeping with previous results, LPS-mediated induction of genes such as *Ifnb1*, *Cxcl9*, or *Il12b* was inhibited upon PGE_2_ coadministration *in vivo* (Figure 3B). These data establish broad functional conservation across cell types and species of the immune-modulatory effects of PGE_2_ in models of *ex vivo* or *in vivo* costimulation.

### PGE_2_-mediated interference with inflammatory gene expression is phenocopied by cyclic AMP but is CREB independent

We next set out to dissect how PGE_2_ controls inflammatory gene expression. PGE_2_ did not alter LPS-induced signaling because IκBα degradation, nuclear translocation of NF-κB p65, internalization of Toll-like receptor 4 (TLR4), phosphorylation of TBK1, and nuclear translocation of IRF3 were preserved in costimulated BMDMs (Figures 3C–3E, S3E, and S3F). Instead, PGE_2_ triggered rapid and transient accumulation of the second messenger cyclic adenosine monophosphate (cAMP) in BMDMs (Figure 3F). Elicitation of cAMP signaling by an activator of adenylyl cyclase (forskolin), a cell-permeable analog of cAMP (dibutyryl-cAMP), or a protein kinase A (PKA) agonist (6-Bnz-cAMP) phenocopied the suppressive effects of PGE_2_ on *Ifnb1* and selected sensitive genes (Figure 3G). The latter genes were also induced defectively in BMDMs exposed to biologically unrelated cAMP-eliciting agents: a selective agonist of the β2-adrenergic receptor (β2AR) or of the adenosine 2B receptor (A2BR) (Figures 3H and 3I). These data suggest that rapid activation of cAMP-PKA signaling by microenvironmental signals underlies selective control of inflammation. We next investigated the role of the cAMP response element-binding protein (CREB) TF, a central PKA target (Altarejos and Montminy, 2011), in PGE_2_-mediated suppression. Rapid CREB1 phosphorylation was observed in BMDMs treated with PGE_2_ as well as with LPS+PGE_2_ (Figure S3G). However, CRISPR-Cas9-mediated targeting of CREB1 in BMDMs (Figure S3H) had no detectable effect on PGE_2_-mediated suppression of inflammatory gene expression, as revealed by qRT-PCR (Figure S3I).

### PGE_2_ functionally inactivates a set of poorly permissive inflammatory gene enhancers

**Figure 4:**
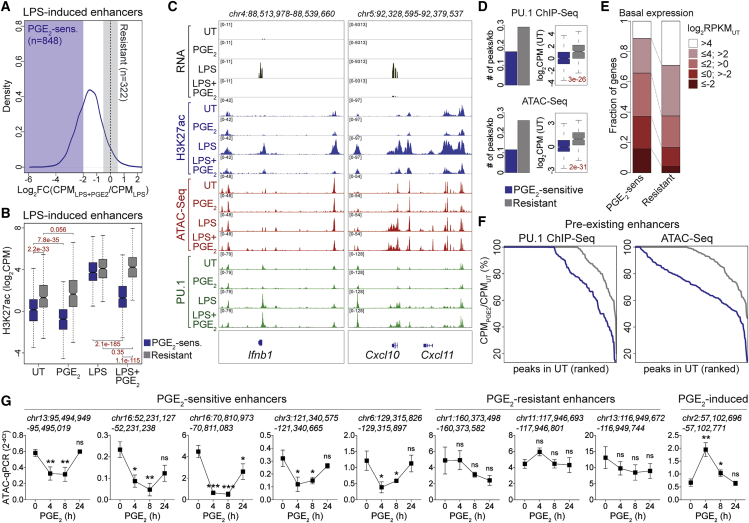
PGE_2_ functionally inactivates a set of poorly permissive inflammatory gene enhancers (A) Density plot showing the effect of PGE_2_ costimulation on LPS-induced H3K27ac. A dotted line indicates lack of effect of the costimulation; blue or gray shaded areas indicate values used to define PGE_2_-sensitive or -resistant enhancers, respectively. Data are from two biological replicates. Pearson correlation > 0.94 for all replicates. (B) H3K27ac ChIP-seq mean signal intensity within PGE_2_-sensitive (blue) or -resistant enhancers (gray) under the indicated conditions. Numbers indicate p values (Mann-Whitney *U* test) for the indicated comparisons. Data are from two biological replicates. Pearson correlation > 0.94 for all replicates. (C) Integrative Genome Viewer (IGV) snapshots showing read coverage of the indicated datasets at selected PGE_2_-sensitive genomic loci in costimulated BMDMs. (D) Number of PU.1 ChIP-seq (top) or ATAC-seq (bottom) peaks per kilobase (bar plot, left) or respective signal intensities (boxplots, right) within PGE_2_-sensitive (blue) or -resistant enhancers (gray). Data refer to UT BMDMs. Numbers indicate p values (Mann-Whitney *U* test) for the indicated comparisons. (E) Stacked bar plot showing the fraction of PGE_2_-sensitive or -resistant genes within the basal expression values (namely, in unstimulated BMDMs) indicated in the legend. (F) Line plots showing PU.1 ChIP-seq or ATAC-seq signal intensities as percent ratio in BMDMs treated with PGE_2_ to UT controls. Data refer to pre-existing open chromatin regions (OCRs) within PGE_2_-sensitive (blue) or -resistant (gray) enhancers. (G) ATAC-qPCR analysis (calculated as 2^-dCt^ over a constitutively open region) of selected PGE_2_-sensitive and -resistant and PGE_2_-induced enhancers in BMDMs stimulated with PGE_2_ for the indicated times. Line plots represent mean ± SD. Data are from three biological replicates. ^∗∗∗^p < 0.001, ^∗∗^p < 0.01, ^∗^p < 0.05 (unpaired t test). See also Figure S4.

The limited effect on TLR4 signaling suggested that PGE_2_ may act at the epigenomic level. We thus performed chromatin immunoprecipitation coupled to sequencing (ChIP-seq) for histone H3 acetylation of lysine 27 (H3K27ac) and PU.1 as well as assay for transposase-accessible chromatin using sequencing (ATAC-seq) experiments. Impaired deposition of H3K27ac was observed at hundreds (n = 848) of LPS-inducible enhancers in costimulated BMDMs (STAR Methods; Figures 4A and 4B; Table S3), exemplified by those at the *Ifnb1* and *Cxcl10* loci (Figures 4C and S4A; Table S3). Luciferase reporter assays confirmed the suppressive effect of PGE_2_ on selected sensitive elements (Figure S4B). In comparison with a set of resistant regions (n = 322) (Figures 4A–4C and S4A; Table S3), PGE_2_-sensitive enhancers had non-permissive chromatin organization with low H3K27ac, H3K4me1, PU.1, and ATAC-seq signals in unstimulated cells (Figures 4B–4D and S4C). This observation was mirrored by a poor basal expression of PGE_2_-sensitive genes (Figures 4E and S4D); low enrichment of H3K4me3, H3K27ac, PU.1, and ATAC-seq at their promoters (Figure S4E; data from Cuartero et al., 2018; Ostuni et al., 2013; Treger et al., 2019); and high sensitivity to bromodomain inhibitors of BET as well as of CBP and p300 (Figures S4F and S4G; Table S1). Similar findings were made for IL-10- or IL-4-sensitive genes (Figures S4D–S4G), indicating that the association with poorly permissive regulatory elements and the requirement for chromatin remodeling underlies vulnerability to antagonism. Notably, treatment of BMDMs with PGE_2_ was sufficient to elicit a further reduction of H3K27ac at sensitive enhancers (Figure 4B; Table S3). PGE_2_-mediated loss of H3K27ac occurred at sensitive enhancers with pre-existing or *de novo* open chromatin regions (STAR Methods; Figure S4I), but it was associated with persistent reduction of chromatin accessibility (up to 8 h) and PU.1 binding only at pre-existing sites (Figures 4F, 4G, S4I, and S4J). We conclude that PGE_2_ drives functional inactivation of a set of poorly permissive enhancers, limiting full induction of the latter in response to inflammatory insults. In keeping with this notion, PGE_2_ treatment caused a significant decrease in basal expression of sensitive genes (Figures 1A, 2E, and S4H), and this behavior was particularly evident for *Ifnb1* and IFN I-dependent transcripts (Figure S1I).

### AP-1 or IRF TFs mark resistant or *de novo* PGE_2_-sensitive enhancers, respectively

**Figure 5:**
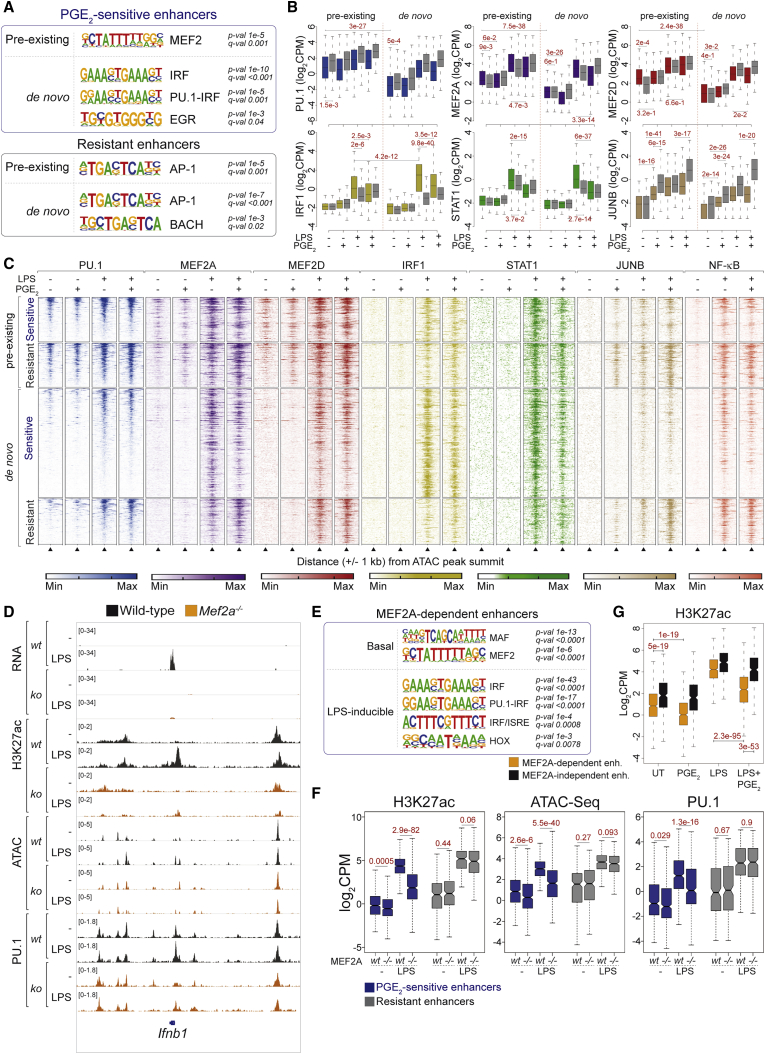
MEF2A controls PGE_2_-sensitive inflammatory gene enhancers (A) Motif enrichment analysis showing top-ranking motifs identified within pre-existing or *de novo* OCRs at PGE_2_-sensitive or resistant enhancers. Putative cognate TF families and associated p values and q values are shown. (B) ChIP-seq signal intensities for the indicated TFs at pre-existing or *de novo* OCRs within PGE_2_-sensitive or -resistant enhancers under the indicated conditions. Numbers denote p values (Mann-Whitney *U* test) for the indicated comparisons. (C) Heatmap showing the intensity of ChIP-seq signals for the indicated TFs under the indicated conditions at pre-existing or *de novo* OCRs within PGE_2_-sensitive or -resistant enhancers. Signal intensities are represented over a 2-kb genomic region spanning the ATAC-seq peak summit. Legends are shown at the bottom. (D) IGV snapshot showing read coverage of the indicated datasets at the *Ifnb1* genomic locus in WT (black) or *Mef2a*^*−/−*^ (orange) iMacs under the indicated experimental conditions. (E) Motif enrichment analysis showing top-ranking motifs identified within basal or LPS-inducible MEF2A-dependent enhancers. Putative cognate TF families and associated p values and q values are shown. (F) Boxplot showing mean intensity values within PGE_2_-sensitive (blue) or resistant (gray) enhancers in the indicated datasets, obtained in WT or MEF2A-deficient iMacs for the indicated conditions. Data are from two biological replicates. Pearson correlation > 0.82 for all replicates. Numbers denote p values (Mann-Whitney *U* test) for the indicated comparisons. (G) H3K27Ac ChIP-seq signal intensities within MEF2A-dependent (orange) and -independent (black) enhancers. Data are from two biological replicates. Pearson correlation > 0.94 for all replicates. Numbers denote p values (Mann-Whitney *U* test) for the indicated comparisons. See also Figure S5.

We next performed motif enrichment analyses to identify sequence determinants of resistance or vulnerability to antagonism by PGE_2_. A clear over-representation of AP-1 TF binding sites (TFBSs) was observed at resistant enhancers (Figure 5A; Table S4). JUNB ChIP-seq experiments validated this prediction because treatment of BMDMs with LPS+PGE_2_ led to increased JUNB occupancy of resistant enhancers (Figures 5B, 5C, and S5A; Table S4) and phosphorylation of the upstream kinases p38 and JNK (Figure S5B). These data suggest that PGE_2_-mediated JUNB activation sustains the activity of a set of inflammatory gene enhancers, in line with the capacity of AP-1 TF to establish chromatin accessibility at bound sites (Biddie et al., 2011). We also observed higher occupancy of resistant enhancers by NF-kB p65 in LPS-stimulated BMDMs (Figure S5C); these observations reflect the binding preference of NF-κB TFs for sites of pre-accessible chromatin and are consistent with inclusion of regulators of the latter pathway within resistant genes. In contrast, *de novo* PGE_2_-sensitive enhancers were enriched in IRF and PU.1:IRF TFBSs (Figure 5A; Table S4), and they were bound strongly by IRF1 in response to LPS (Figures 5B and 5C; Table S4). PGE_2_ treatment selectively suppressed IRF1 as well as STAT1 occupancy of sensitive enhancers (Figures 5B and 5C; Table S4), likely reflecting the IFN-β synthesis defect in costimulated BMDMs. Addition of exogenous IFN-β was indeed sufficient to restore H3K27ac at selected sensitive loci (Figure S5D).

### MEF2 TFs mark pre-existing sensitive enhancers in unstimulated macrophages

When focusing on pre-existing PGE_2_-sensitive enhancers, we detected over-representation of the 5′-CTATTTTTGG-3′ motif, corresponding to the myocyte enhancer factor 2 (MEF2) TFBS (Figure 5A; Table S4). ChIP-seq analyses revealed broad basal genome occupancy by MEF2A and MEF2D (26,472 and 18,709 peaks, respectively) in macrophages, with MEF2C binding to fewer sites (6,340 peaks) (Figure S5E). MEF2A binding sites occurred within poised or active promoters and enhancers (Figure S5F) and largely overlapped with peaks of the lineage-determining TFs PU.1, IRF8, and C/EBPβ (Figure S5G). We next assessed the distribution of MEF2 TFs at PGE_2_-regulated inflammatory gene enhancers. Notably, binding of MEF2A and MEF2D at pre-existing sensitive enhancers was evident already in unstimulated BMDMs (Figures 5B and 5C), in line with selective enrichment of the MEF2 TFBS at the latter regions (Figure 5A). Weaker basal MEF2 binding at resistant enhancers was, however, observed (Figures 5B and 5C; Table S4), possibly reflecting occupancy via protein-protein interactions. LPS treatment of macrophages elicited an increase in MEF2 ChIP-seq signals at all sets of enhancers, which was affected significantly by PGE_2_ at sensitive sites (Figures 5B and 5C; Table S4). These data highlight MEF2 TFs as candidate regulators of the macrophage *cis*-regulatory landscape. More specifically, sequence-specific MEF2 marking of pre-existing PGE_2_-sensitive enhancers may be required for their subsequent activation in response to inflammatory signals.

### MEF2A controls the basal and LPS-inducible enhancer landscape of macrophages

To dissect the epigenomic actions of MEF2, we generated immortalized macrophages (iMacs) (Redecke et al., 2013; Roberts et al., 2019; Figure S5H) bearing single or combined *Mef2a*, *Mef2c*, or *Mef2d* edits (Table S7). *Mef2a*^*−/−*^ iMacs did not show defects in viability (Figure S5I), proliferation, or differentiation but failed to upregulate *Ifnb1* upon LPS treatment (Figure S5J), leading us to focus on this TF. Genomic analyses of *Mef2a*^*−/−*^ iMacs revealed profound alterations of the *cis*-regulatory repertoire, with hundreds of enhancers losing H3K27ac in UT (n = 998) or LPS-treated (n = 981) macrophages (STAR Methods; Figure S5K; Table S5). Notably, loss of MEF2A caused functional inactivation and defective induction of regulatory elements of the *Ifnb1* locus (Figure 5D). Compared with a set of control regions (STAR Methods), MEF2A-dependent basal enhancers were enriched in MAF and MEF2 TFBSs, underscoring direct control via sequence-specific genome marking (Figure 5E; Table S5). LPS-inducible MEF2A-dependent enhancers were instead enriched in IRF and PU.1:IRF sites (Figure 5E; Table S5), consistent with defective chromatin dynamics at the *Ifnb1* locus in *Mef2a*^−/−^ cells. We next investigated the reciprocal effect of MEF2A and PGE_2_ on macrophage *cis*-regulatory elements. MEF2A loss caused a reduction of basal and LPS-inducible histone acetylation, chromatin accessibility, and PU.1 binding at PGE_2_-sensitive enhancers (Figure 5F; Table S5). Reciprocally, MEF2A-dependent enhancers induced by LPS displayed low basal H3K27ac and were heavily affected by PGE_2_ treatment (Figure 5G; Table S5). Our data uncover MEF2A as a regulator of the PGE_2_-sensitive enhancer landscape in macrophages and highlight functional antagonism between PGE_2_ and MEF2A. Deficiency of MEF2A or PGE_2_ treatment targeted sensitive enhancers such as those of the *Ifnb1* locus, leading to defective IFN-β synthesis and defective secondary activation of IRF-dependent elements.

### MEF2A is required for IFN I induction in macrophages treated with LPS

**Figure 6:**
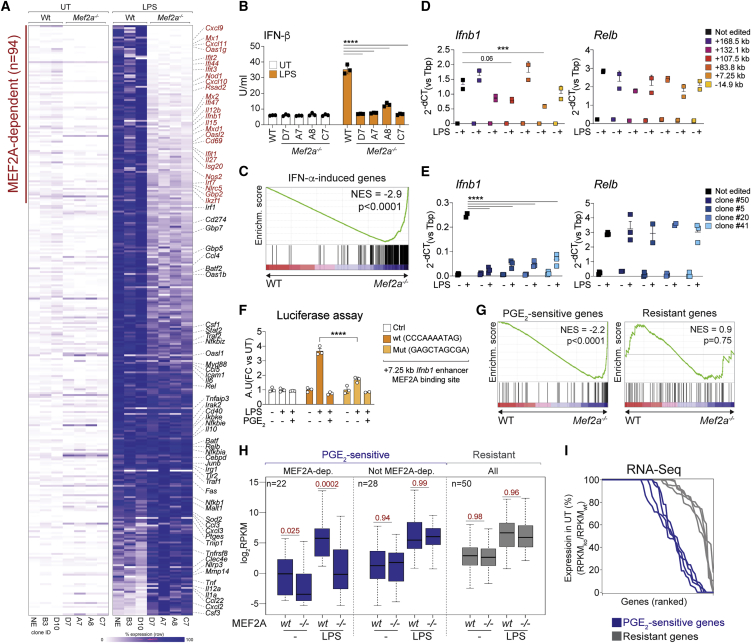
MEF2A controls inflammatory gene expression in macrophages (A) Heatmap showing the behavior of LPS-inducible genes (n = 312; STAR Methods) in WT or *Mef2a*^*−/−*^ macrophages. The defined set of MEF2A-dependent genes (n = 94; STAR Methods) is highlighted in red. Colors represent row-normalized percentages of gene expression. Selected gene names are shown on the right, and color legends and clone IDs are shown at the bottom. Data are from three biological replicates. Pearson correlation > 0.93 for all replicates. (B) IFN-β release by WT or *Mef2a*^*−/−*^ iMacs stimulated with LPS. Genotypes and IDs of the individual clones are shown. The bar plot represents mean ± SD. Data are from three biological replicates. ^∗∗∗∗^p < 0.0001 (two-way ANOVA). (C) Gene set enrichment analysis (GSEA) of IFN-α-induced genes (gene set) in ranked gene lists obtained by comparing LPS-stimulated *Mef2a*^*−/−*^ versus WT iMacs. Normalized enrichment score (NES) and p value are shown. (D) qRT-PCR analysis of *Ifnb1* and *Relb* expression in BMDMs upon CRISPR-Cas9 targeting of the indicated putative *Ifnb1* enhancers, named according to their distance to the transcription start site (TSS). Dot plots represent mean ± SD. Data are from three biological replicates. Numbers indicate p values for the corresponding comparisons (two-way ANOVA). (E) qRT-PCR analysis of *Ifnb1* and *Relb* expression by iMac clones upon CRISPR-Cas9-mediated editing of the MEF2A binding site within the +7.25-kb *Ifnb1* enhancer. Dot plots represent mean ± SD. Data are from three biological replicates. Numbers indicate p values for the corresponding comparisons (two-way ANOVA). (F) Luciferase reporter activity of the +7.25-kb *Ifnb1* enhancer upon mutagenesis of the MEF2A TFBs in BMDMs treated with LPS or LPS+PGE_2_. a.u., arbitrary unit. The bar plot represents mean ± SD. Data are from three biological replicates. Numbers indicate p values for the corresponding comparisons (two-way ANOVA). (G) GSEA of PGE_2_-sensitive or -resistant genes (gene sets) in ranked gene lists obtained comparing LPS-stimulated *Mef2a*^*−/−*^ versus WT iMacs. NES and p value are shown for each plot. (H) Boxplot showing mean expression values of PGE_2_-sensitive (MEF2A-dependent or not MEF2A-dependent STAR Methods) or resistant genes in WT or MEF2A-deficient iMacs under the indicated conditions. Data are from three biological replicates. Pearson correlation > 0.93 for all replicates. Numbers indicate p values for the corresponding comparisons (Mann-Whitney *U* test). (I) Line plot showing expression of PGE_2_-sensitive or -resistant genes as percent ratio of unstimulated MEF2A-deficient versus WT iMacs. Lines represent individual MEF2A-deficient clones. See also Figure S6.

We next elucidated the contribution of MEF2 TFs to inflammatory gene expression by RNA-seq. Although loss of MEF2C and/or of MEF2D had a minor effect (Table S6), we observed profoundly impaired responses to LPS in *Mef2a*^*−/−*^ iMacs, with defective induction of a large set of transcripts (n = 94 of 312), such as *Ifnb1*, *Il12b*, or *Cxcl10* (Figure 6A; Table S6). In line with our previous analyses, *Mef2a*^*−/−*^ iMacs failed to release IFN-β upon LPS (Figure 6B), and MEF2A-dependent genes were enriched in IFN I-stimulated transcripts (Figure 6C). The transcriptional defect of LPS-stimulated *Mef2a*^*−/−*^ iMacs could be at least partially restored by addition of recombinant IFN-β, as measured by qRT-PCR on selected MEF2A-dependent genes (Figure S6A). MEF2A loss did not affect IκBα degradation, nuclear translocation of NF-κB p65, internalization of TLR4, or phosphorylation of TBK1 upon LPS treatment (Figures S6B–S6D). Furthermore, *Mef2a*^*−/−*^ iMacs did not release more IL-10 or PGE_2_ in response to LPS than control cells (Figure S6E), and defective inflammatory gene expression in cells of the latter genotype was not restored by IL-10R blockade (Figure S6F). We next performed CRISPR-Cas9-mediated targeting of six putative *Ifnb1* enhancers to determine whether MEF2A directly controlled gene transcription (Figure S6G). Disruption of the MEF2A binding site at the +7.25-kb enhancer led to defective *Ifnb1* induction in a bulk population of BMDMs (Figure 6D), and this finding was confirmed by analyses of clones of iMacs bearing homogeneous and validated edits (Figures 6E and S6H; Table S7). The latter region displayed PGE_2_-sensitive MEF2A occupancy (Figure S6G) as well as PGE_2_-sensitive and MEF2A-dependent H3K27ac (Figures S6I and S6J). Luciferase assays in macrophages revealed that reporter activity of the wild-type +7.25-kb *Ifnb1* enhancer sequence was stimulated by LPS and suppressed by PGE_2_ (Figure 6F), whereas site-specific mutagenesis of the MEF2A binding site abolished activity in LPS-treated macrophages (Figure 6F). Collectively, these data highlight MEF2A as a direct regulator of IFN I induction in LPS-treated macrophages.

### MEF2A controls basal and inducible expression of a set of PGE_2_-sensitive genes

Integrated analyses of RNA-seq datasets highlighted a key role of MEF2A in the control of PGE_2_-sensitive inflammatory gene expression. The overlap between PGE_2_-sensitive and MEF2A-dependent genes was significantly higher than expected by chance, whereas the overlap between resistant genes and MEF2A-dependent genes was significantly lower than expected by chance (Figure S6K). Gene set enrichment and reciprocal analyses of RNA-seq datasets revealed that genes antagonized by PGE_2_ were enriched in MEF2A-dependent transcripts, whereas resistant controls were not (Figures 6G and S6K). Furthermore, a set of PGE_2_-sensitive genes showed defective expression in *Mef2a*^−/−^ macrophages in unstimulated and LPS-treated cells (Figures 6H, 6I, and S6L). Basal and LPS-induced expression of MEF2A-dependent genes was instead selectively antagonized by PGE_2_ (Figure S6M). Together with previous observations, our data highlight an antagonistic PGE_2_-MEF2A axis controlling macrophage activation. MEF2A deletion and PGE_2_ treatment caused loss of basal and LPS-inducible expression of a set of sensitive genes enriched in IFN I-regulated transcripts. The overlap between PGE_2_-sensitive and MEF2A-dependent genes was, however, not complete, highlighting the existence of PGE_2_-independent activities of MEF2A and vice versa.

### MEF2A controls IFN I induction in response to nucleic acids or microbial pathogens

**Figure 7:**
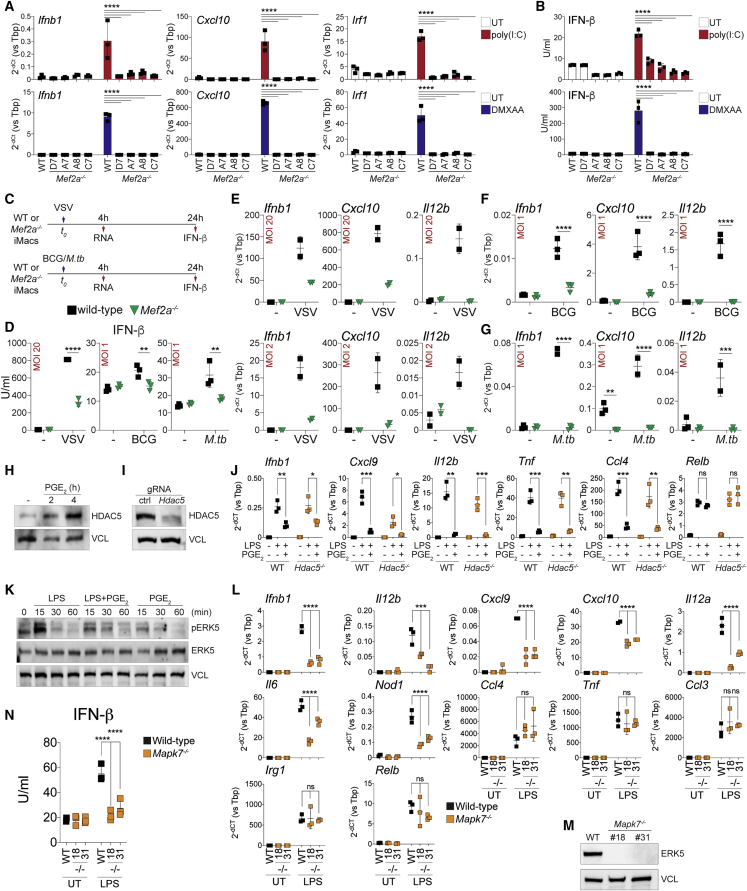
MEF2A controls IFN I induction by nucleic acids or pathogens and is targeted by PGE_2_ partly via ERK5 (A) qRT-PCR analysis of *Ifnb1*, *Cxcl10*, or *Irf1* in WT or *Mef2a*^*−/−*^ iMacs stimulated for 4 h with poly(I:C) (red, top) or DMXAA (blue, bottom). Genotypes and IDs of the individual clones are shown. Bar plots represent mean ± SD. Data are from three biological replicates. ^∗∗∗∗^p < 0.0001 (two-way ANOVA). (B) IFN-β release by WT or *Mef2a*^*−/−*^ iMacs stimulated with poly(I:C) (red, top) or DMXAA (blue, bottom). Genotypes and IDs of the individual clones are shown. Bar plots represent mean ± SD. Data are from three biological replicates. ^∗∗∗∗^p < 0.0001 (two-way ANOVA). (C) Experimental layout of *in vitro* infection of iMacs with VSV, BCG, or *M.tb*. (D) IFN-β release by WT (black) or *Mef2a*^*−/−*^ (green) iMacs infected with VSV, BCG, or *M.tb*. MOI values are reported for each plot. Dot plots represent mean ± SD. Data are from two or three biological replicates. ^∗∗∗∗^p < 0.0001, ^∗∗^p < 0.005 (two-way ANOVA test). (E–G) qRT-PCR analysis of *Ifnb1*, *Cxcl10*, or *Il12b* in WT (black) or *Mef2a*^*−/−*^ (green) iMacs infected with VSV (E), BCG (F), or *M.tb* (G). MOI values are reported for each plot. Dot plots represent mean ± SD. Data are from two or three biological replicates. ^∗∗∗∗^p < 0.0001, ^∗∗∗^p < 0.001 (two-way ANOVA). (H and I) Western blot analyses of HDAC5 and loading control (VCL) in BMDMs stimulated with PGE_2_ for the indicated times (H) or in BMDMs upon CRISPR-Cas9-mediated targeting of *Hdac5* (I). (J) qRT-PCR analysis of PGE_2_-sensitive and -resistant genes in WT (black) or HDAC5-deficient (orange) BMDMs stimulated as indicated. Dot plots represent mean ± SD. Data from three biological replicates. ^∗∗∗^p < 0.001, ^∗∗^p < 0.01, ^∗^p < 0.05 (unpaired t test). (K) Western blot analyses for phosphorylated ERK5 (Thr218/Tyr220) as well as ERK5 and VCL as loading controls in BMDMs stimulated as indicated. (L) qRT-PCR analysis of PGE_2_-sensitive and resistant genes in WT (black) or *Mapk7*^*−/−*^ (orange) iMacs clones stimulated as indicated. Genotypes and IDs of the individual clones are shown. Dot plots represent mean ± SD. Data are from three biological replicates. ^∗∗∗∗^p < 0.0001, ^∗∗∗^p < 0.001 (unpaired t test). (M) Western blot analyses of ERK5 and VCL in WT and *Mapk7*^*−/−*^ iMacs clones. Genotypes and IDs of the individual clones are shown. (N) IFN-β release by WT (black) and *Mapk7*^*−/−*^ (orange) iMacs stimulated as indicated. Dot plot represents mean ± SD. Data are from three biological replicates. ^∗∗∗∗^p < 0.0001 (two-way ANOVA). See also Figure S7.

We wanted to determine whether loss of MEF2A would affect responses to other innate immune stimuli. Treatment with poly(I:C), CpG-DNA or 5,6-dimethylxanthenone-4-acetic acid (DMXAA), synthetic activators of TLR3, TLR9, or cyclic guanosine monophosphate (GMP)-AMP synthase (cGAS), respectively, stimulated inflammatory gene expression and IFN-β synthesis in wild-type (WT) but not *Mef2a*^*−/−*^ iMacs (Figures 7A, 7B, and S7A). Defective transcriptional induction of IFN I (Figures S7B–S7E) and deposition of H3K27ac at PGE_2_-sensitive enhancers (Figure S7F) were also observed in BMDMs stimulated with DMXAA or poly(I:C) in the presence of PGE_2_ or forskolin. We next assessed the effect of MEF2A deficiency or PGE_2_ administration on macrophage responses to viral or bacterial pathogens, such as vesicular stomatitis virus (VSV), *M. bovis* Bacillus Calmette-Guérin (BCG), or *Mycobacterium tuberculosis* (*M.tb*) (Figure 7C). Potent transcript and protein induction of IFN-β as well as expression of inflammatory response genes was observed in WT cells, whereas *Mef2a*^*−/−*^ iMacs (Figures 7D–7G) or WT BMDMs treated with PGE_2_ (Figures S7G and S7H) showed impaired responses to VSV, BGC, or *M.tb*. Thus, MEF2A and PGE_2_ reciprocally control inducible IFN I synthesis and inflammatory gene expression in response to multiple innate immune stimuli and microbial pathogens.

### IL-10 targets inflammatory gene expression at least partially via STAT3

Analogous to PGE_2_, IL-10 treatment did not clearly affect TLR4 signaling or internalization (Figures S7I–S7L) and was sufficient to suppress basal transcription of the respective sensitive genes (Figure S7M). Genomic analyses in costimulated BMDMs identified a large number (n = 1,093) of IL-10-sensitive inflammatory gene enhancers (Figure S7N; Table S3). The latter regions displayed poorly permissive basal chromatin organization and partially overlapped with PGE_2_-sensitive enhancers (Figures S7O–S7Q; Table S3). IL-10 treatment caused a significant reduction of basal H3K27ac at sensitive enhancers (Figure S7O), whereas chromatin accessibility and PU.1 binding remained largely unaffected (Figure S7P). These observations indicate that IL-10 has detectable capacity to functionally inactivate regulatory elements of antagonistic programs, in line with reports showing suppression of LPS-inducible histone acetylation (Conaway et al., 2017); this capacity was, however, limited compared with what was observed for PGE_2_. Motif analyses identified over-representation of IRF and PU.1:IRF, but not of MEF2 sites, at pre-existing or *de novo* IL-10-sensitive enhancers (Figure S7R; Table S4). Neither enhancers targeted selectively by IL-10 nor those commonly sensitive to PGE_2_ and IL-10 were enriched in MEF2 sites but, rather, contained IRF TFBSs (Figure S7S; Table S4). We conclude that sequence-specific control by MEF2A is restricted to PGE_2_-sensitive enhancers and that epigenomic suppression by IL-10 occurs at IRF-regulated sites via an alternative mechanism. In this context, antagonism by IL-10 of LPS-mediated induction of *Ifnb1*, *Il12b*, and *Il6* (among other sensitive genes) was lost in costimulated *Stat3*^*−/−*^ BMDMs (Figure S7T).

### PGE_2_ targets MEF2A-dependent inflammatory gene expression partly via ERK5

We next investigated the mechanistic link between PGE_2_ and MEF2A. Our ChIP-seq showed that basal MEF2A occupancy at pre-existing sensitive enhancers was slightly but significantly affected by PGE_2_ treatment (Figures 5B and 5C). These observations prompted us to hypothesize that PGE_2_ could target transcriptional coregulators of MEF2A. Class IIa histone deacetylases (HDACs) (i.e., HDAC4, HDAC5, HDAC7, and HDAC9) are known MEF2 interactors that, upon signal-dependent nuclear shuttling, suppress transcription (Haberland et al., 2009) and have been implicated previously in inflammatory gene expression control (Guerriero et al., 2017; Luan et al., 2014). We focused on HDAC5 because it was expressed basally in BMDMs and induced further by PGE_2_ treatment (Figure 7H); disruption of *Hdac5* in BMDMs was, however, not sufficient to restore LPS-driven expression of PGE_2_-sensitive genes in costimulated cells (Figures 7I and 7J). Although compensatory effects cannot be excluded, the data do not support a prominent role of class IIa HDACs in this setting. We next focused on extracellular-signal-regulated kinase (ERK) 5, an atypical mitogen-activated protein kinase (MAPK) known to interact directly with MEF2 via a C-terminal transactivating domain (Pereira and Rodrigues, 2020) and to mediate context-dependent actions in myeloid cells (Giurisato et al., 2018; Luiz et al., 2020). In previous studies, cAMP-PKA signaling has been shown to inhibit growth factor-mediated activation of ERK5 (Pearson et al., 2006). Rapid and transient phosphorylation of ERK5 was observed in macrophages treated with LPS, but the latter modification was reduced by PGE_2_ coadministration (Figure 7K). We thus assessed the role of ERK5 in inflammatory gene expression. LPS-stimulated *Mapk7*^*−/−*^ iMacs (Table S7) showed reduced expression of a set of PGE_2_-sensitive genes, including *Ifnb1*, *Il12b*, and *Cxcl9*, but not of resistant controls (Figures 7L and 7M). The observed transcriptional defects were also associated with impaired IFN-β synthesis in ERK5-deficient cells (Figure 7N). These data indicate that PGE_2_ suppresses inflammatory gene expression, at least in part, by antagonizing LPS-mediated activation of ERK5, a known transcriptional partner of MEF2.

## Discussion

In this study, we set out to define how inflammatory gene expression is controlled by antagonistic signals. An integrated genomic analysis of costimulated macrophages uncovered specific and shared suppressive effects of PGE_2_, IL-10, and IL-4 on LPS-inducible transcription. In particular, PGE_2_ blocked IFN-β induction upon *ex vivo* or *in vivo* treatment with LPS, and it boosted IL-10 secretion by costimulated macrophages. Overall, genes sensitive to costimulation displayed analogous genomic properties: enrichment in IFN I-regulated transcripts, low basal expression, association with poorly accessible promoters, and high sensitivity to inhibitors of chromatin remodeling. Such a stringent genomic context ensures tightly regulated transcription upon LPS exposure (Nicodeme et al., 2010; Seeley et al., 2018). Our data indicate that the non-permissive chromatin organization at inducible genes also imparts higher vulnerability to antagonistic signals, enabling context-dependent control of inflammation. This notion is reinforced by the finding that PGE_2_ and IL-10 negatively targeted sets of LPS-inducible enhancers with low basal histone acetylation, chromatin accessibility, and PU.1 occupancy in the absence of detectable interference with TLR4 signaling. Together with those of others (Czimmerer et al., 2018; Kang et al., 2017; Piccolo et al., 2017; Qiao et al., 2016), our data establish chromatin as the main integration platform for antagonistic signals in macrophages.

A key observation was that treatment of macrophages with PGE_2_ elicited loss of basal expression of IFN I-dependent genes and of histone acetylation at sensitive enhancers, leading to impaired induction upon LPS treatment. We also provide evidence of IL-10-mediated functional inactivation of sensitive regulatory elements, mirroring a decrease in basal expression of antagonized genes. These findings complement data showing that IFN-γ promotes disassembly of tissue repair gene enhancers in human macrophages (Kang et al., 2017). Reciprocal suppression of enhancers of conflicting programs thus ensures mutual exclusion of incompatible biological properties and preserves the identity of costimulated cells. On the other hand, groups of genes induced by antagonistic pathways, such as those triggered by LPS or PGE_2_, can co-exist in single cells, as also shown recently (Muñoz-Rojas et al., 2021). The “mixed” phenotypes of costimulated macrophages are incompatible with dogmatic definitions of polarization states. Rather, they may exemplify the diversity of macrophages in settings such as cancer, where immune suppressive and stimulatory signals co-exist (Kratochvill et al., 2015; Murray et al., 2014; Osborne et al., 2014).

MEF2 TFs control cardiomyocyte, smooth muscle, and neuronal development (Potthoff and Olson, 2007), but their roles in the innate immune system are poorly characterized. A study using flies found increased susceptibility to infection upon fat-body-specific loss of MEF2 (Clark et al., 2013). We identified MEF2A as a non-redundant regulator of the inflammatory epigenome of macrophages and, more specifically, of PGE_2_-sensitive enhancers. Deletion of MEF2A caused functional inactivation of the latter regions and prevented induction of IFN-β upon exposure to innate immune stimuli or pathogens. Mechanistically, PGE_2_ interfered with LPS-mediated activation of ERK5, a known transcriptional partner of MEF2 (Pereira and Rodrigues, 2020), whose deletion led to defective IFN-β synthesis. The genomic consequences of MEF2A loss and PGE_2_ treatment overlapped broadly, supporting a functional connection in macrophages. However, PGE_2_-independent activities of MEF2A (and vice versa) were also evident and are a matter of future investigation. We hypothesize that MEF2A acts in a context-dependent manner through a dynamic network of interactors. On the other hand, it will be of interest to dissect how the complex immunological properties of PGE_2_, including its well-known proinflammatory effects (Dennis and Norris, 2015), are linked to MEF2A activity.

MEF2 TFs are highly expressed in tissue-resident macrophages, such as brain microglia (Geirsdottir et al., 2019; Lavin et al., 2014), and there is evidence of involvement of MEF2C (Deczkowska et al., 2017) and aberrant PGE_2_ activity (Johansson et al., 2013; Minhas et al., 2021) in age-associated neuroinflammation. Our findings raise the possibility that a PGE_2_-MEF2 axis controls the physiopathological activity of macrophages *in vivo*. Although we cannot exclude a role of alternative downstream pathways, the observation that PGE_2_-mediated immune modulation was phenocopied by cAMP is particularly intriguing. Multiple regulators of tissue immune homeostasis indeed elicit cAMP; β-adrenergic signaling in a population of intestinal macrophages limits local inflammation (Gabanyi et al., 2016; Matheis et al., 2020), adenosine protects hematopoietic stem cells from oxidative stress and suppresses allograft rejection (Hirata et al., 2018), lactic acid polarizes tumor-associated macrophages toward an immune-regulatory phenotype via cAMP (Bohn et al., 2018; Colegio et al., 2014), and some bacterial toxins with adenylyl cyclase activity elicit suppressive cAMP signaling in macrophages (Agarwal et al., 2009; Leppla, 1982). Thus, it is tempting to speculate that the immune-modulatory actions of diverse tissue signals may converge on cAMP-mediated suppression of IFN I responses.

Accumulating evidence indicates that targeting PGE_2_ may improve the efficacy of cancer immunotherapy by unleashing inflammatory gene expression in tumor-infiltrating immune cells (Hou et al., 2016). Inhibition of cyclooxygenase-2 (COX-2) in a mouse model of melanoma stimulated tumor rejection, driven by IFN I production by innate immune cells, and increased natural killer cell recruitment and dendritic cell activation (Bonavita et al., 2020; Böttcher et al., 2018; Zelenay et al., 2015). Identification of MEF2A as an essential transcriptional regulator of IFN I synthesis that is targeted negatively by PGE_2_ may pave the way for development of anti-cancer agents. More generally, our study raises the possibility that targeting MEF2A-dependent inflammatory gene expression by cAMP-eliciting environmental cues may be a general mechanism linking tissue homeostasis and immune control, which may become aberrant during tumor progression or microbial infection.

### Limitations of study

The use of a reductionist model—namely, costimulation of BMDMs and iMacs—enabled systematic analysis of the interplay between immune-stimulatory and modulatory signals. To what extent our observations mirror the complexity of inflammatory responses in physiology or pathology needs to be validated. For instance, it will be critical to investigate the role of MEF2A in controlling IFN I induction *in vivo* during infection, tissue damage, or cancer. This study provides a description and an initial molecular characterization of a PGE_2_-MEF2A axis in control of inflammatory gene expression. Follow-up studies are needed to fully resolve the transcriptional and epigenomic network underlying the activities of MEF2A in macrophages as well as the inhibitory effects of PGE_2_.

## STAR★Methods

### Key resources table

**Table:** 

REAGENT or RESOURCE	SOURCE	IDENTIFIER
**Antibodies**		

Mouse anti-IKappaB-alpha (L35A5) Monoclonal Antibody	Cell Signaling Technology	Cat# 4814; RRID: AB_390781
Rabbit Polyclonal anti-p38 MAPK Antibody	Cell Signaling Technology	Cat# 9212; RRID: AB_330713
Rabbit Polyclonal anti-SAPK/JNK Antibody	Cell Signaling Technology	Cat# 9252; RRID: AB_2250373
Rabbit anti-IRF-3 (D38B9) Monoclonal Antibody	Cell Signaling Technology	Cat# 4302; RRID: AB_1904036
Rabbit anti-IRF-1 (D5E4) Monoclonal Antibody	Cell Signaling Technology	Cat# 8478; RRID: AB_10949108
Rabbit anti-phospho-p38 MAPK (Thr180/Tyr182) Antibody	Cell Signaling Technology	Cat# 9211; RRID: AB_331641
Rabbit anti-phospho-SAPK/JNK (Thr183/Tyr185) Antibody	Cell Signaling Technology	Cat# 9251; RRID: AB_331659
Rabbit anti-phospho-STAT1 (Tyr701) Antibody	Cell Signaling Technology	Cat# 9171; RRID: AB_331591
Rabbit anti-phospho-STAT2 (Tyr689) Antibody	Sigma-Aldrich	Cat# 07-224; RRID: AB_2198439
Rabbit anti-CREB (48H2) Monoclonal antibody	Cell Signaling Technology	Cat. 9197; RRID_AB_331277
Rabbit anti-phospho-CREB (Ser133) (87G3) Monoclonal antibody	Cell Signaling Technology	Cat. 9198; RRID_AB_2561044
Rabbit anti-HDAC5 (D1J7V) Monoclonal antibody	Cell Signaling Technology	Cat. 20458; RRID_AB_2713973
Rabbit anti-ERK5 (EP791Y) Monoclonal antibody	Abcam	Cat. ab40809
Rabbit anti-phospho-ERK5 (Thr218/Tyr220) Polyclonal antibody	Cell Signaling Technology	Cat. 3371; RRID_AB_2140424
Mouse anti-beta-Actin Monoclonal Antibody, Unconjugated, Clone AC-15	Sigma-Aldrich	Cat# A1978; RRID: AB_476692
Mouse anti-Vinculin Monoclonal Antibody, Unconjugated, Clone v284	Sigma-Aldrich	Cat# 05-386; RRID: AB_309711
Mouse anti-Lamin B1 Monoclonal Antibody, Unconjugated, Clone A-11	Santa Cruz Biotechnology	Cat# sc-377000
Rabbit anti-NF-kappaB p65 (D14E12) XP Monoclonal Antibody	Cell Signaling Technology	Cat# 8242; RRID: AB_10859369
Rabbit anti-phospho-TBK1/NAK (Ser172) (D52C2) XP Monoclonal Antibody	Cell Signaling Technology	Cat# 5483; RRID: AB_10693472
Rabbit Polyclonal Anti-H3K27Ac Antibody	Abcam	Cat# ab4729; RRID: AB_2118291
Rabbit anti-PU.1 (T-21) Polyclonal Antibody	Santa Cruz Biotechnology	Cat# sc-352; RRID: AB_632289
Rabbit anti-MEF2A Monoclonal Antibody, Unconjugated, Clone EP1706Y	Abcam	Cat# ab76063; RRID: AB_1310444
Rabbit Anti-MEF2D Polyclonal Antibody, Unconjugated	Abcam	Cat# ab32845; RRID: AB_776269
Mouse anti-IRF1 (E-4) Monoclonal Antibody	Santa Cruz Biotechnology	Cat# sc-514544-X
Mouse anti-STAT1 (C-136) Monoclonal Antibody	Santa Cruz Biotechnology	Cat# sc-464-X
Rabbit anti-JUNB (N-17) Polyclonal Antibody	Santa Cruz Biotechnology	Cat# sc-46-X
Rabbit anti-NF-kappaB p65 (C-20) Polyclonal Antibody	Santa Cruz Biotechnology	Cat# sc-372-X
Rat anti-mouse CD284 (TLR4) Antibody, PE conjugated	Biolegend	Cat# 145404; RRID: AB_2561874
Rat anti-mouse/human CD11b Antibody, APC conjugated	Biolegend	Cat# 101212; RRID: AB_312795
Rat anti-mouse F4/80 Antibody, PE conjugated	Biorad	Cat# MCA497; RRID: AB_2098196
Rat anti-mouse Ly-6C Monoclonal Antibody (HK1.4), APC-eFluor 780 conjugated	eBioscience, Thermo Fisher Scientific	Cat# 47-5932-82; RRID: AB_2573992
Hamster anti-mouse Cd11c Monoclonal Antibody (HL3), BV421 conjugated	BD Biosciences	Cat# 562782; RRID: AB_2737789
Rat anti-mouse SiglecF (clone E50-2440), PerCP-Cy5.5	BD Biosciences	Cat# 565526; RRID: AB_2739281
Goat anti-rabbit IgG (H+L) Secondary Antibody, Alexa Fluor Plus 488 conjugated	Thermo Fisher Scientific	Cat# A32731; RRID: AB_2633280
Goat anti-mouse IgG (H+L) Secondary Antibody, HRP	Thermo Fisher Scientific	Cat# 62-6520; RRID: AB_2533947
Goat anti-rabbit IgG (H+L) Secondary Antibody, HRP	Thermo Fisher Scientific	Cat# 65-6120; RRID: AB_2533967
Rat Anti-IL-10 Receptor (1B1-3a) Monoclonal antibody	BioXCell	Cat. BE0050; RRID_AB_1107611
Rat Isotype Control (2A3) Monoclonal antibody	BioXCell	Cat. BE0089; RRID_AB_1107769

**Bacterial and virus strains**		

Vesicular Stomatitis Virus (VSV)	Matteo Iannacone	N/A
Bacillus Calmette-Guérin (BCG)	Daniela Maria Cirillo	N/A
*Mycobacterium tuberculosis* (*M.tb*)	Daniela Maria Cirillo	N/A

**Chemicals, peptides, and recombinant proteins**		

Lipopolysaccharide (LPS)	Enzo Life Sciences	ALX-581-010-L001; CAS: 61512-20-7
Prostaglandin E2	Cayman Chemical	CAY-14010; CAS: 363-24-6
Recombinant Mouse Interferon alpha (IFN-α)	PBL Assay Science	12100-1
Recombinant Mouse Interleukin 10 (IL-10)	R&D Systems	417-ML
Recombinant Mouse Interleukin 4 (IL-4)	Peprotech	214-14
5,6-Dimethylxanthenone-4-acetic acid (DMXAA)	Invivogen	TLRL-DMX
Polyinosinic:polycytidylic acid (Poly(I:C))	Invivogen	TLRL-PIC
CpG	Invivogen	TLR-1668
Forskolin	Sigma-Aldrich	F3917-10MG; CAS: 66575-29-9
6-Bnz-cAMP sodium salt	Tocris Bioscience, Bio-Techne	5255; CAS: 1135306-29-4
N^6^,2′-O-Dibutyryladenosine 3′,5′-cyclic monophosphate sodium salt (db-cAMP)	Sigma-Aldrich	D0260-25MG; CAS: 16980-89-5
Salmeterol Xinafoate	Sigma-Aldrich	S5068; CAS: 94749-08-3
BAY60-6583	Sigma-Aldrich	SML1958; CAS: 910487-58-0
PFI-1 (PF-6405761) – BRD2/BRD4i	Selleck Chemicals	S1216; CAS: 1403764-72-6
SGC-CBP30 (CREBBP/EP300i)	Selleck Chemicals	S7256; CAS: 1613695-14-9
Recombinant Mouse Interferon beta (IFN-β)	PBL Assay Science	12401-1
CRISPR-associated protein 9 (Cas9)	In-house produced	N/A
TrueCut Cas9 Protein v2	Thermo Fisher Scientific	A36498
BsmBI	New England Biolabs	R0580
NheI	New England Biolabs	R0131
XhoI	New England Biolabs	R0146
DpnI	New England Biolabs	R0176
Endotoxin-free Plasmid DNA Purification Kit	Macherey-Nagel	740420.50
T7 Endonuclease	New England Biolabs	E3321
AMPure Beads XP	Beckman Coulter Life Science	A63881
Dynabeads Protein G	Thermo Fisher Scientific	10009D
Random Primers	Thermo Fisher Scientific	48190011
PCR Nucleotide Mix	Promega	C1145
Digitonin	Promega	G9441; CAS: 11024-24-1
Proteinase K solution	Promega	MC5005; CAS: 39450-01-6
LIVE/DEAD Fixable Yellow Dead Cell Stain Kit	Thermo Fisher Scientific	L34959
Paraformaldehyde solution	Santa Cruz Biotechnology	sc-281692; CAS: 30525-89-4
DAPI	Sigma-Aldrich	D9542; CAS: 28718-90-3
Aqua-Poly/Mount	Polysciences	18606
Triton X-100 solution	Sigma-Aldrich	93443; CAS: 9002-93-1
Bovine Serum Albumin (BSA)	Sigma-Aldrich	A8806; CAS: 9048-46-8
Phosphate Buffered Saline (PBS)	Corning	21-031-CV
Formaldehyde solution	Sigma-Aldrich	F8775; CAS:50-00-0
HEPES	Sigma-Aldrich	H3375; CAS: 7365-45-9
EDTA	Sigma-Aldrich	E9884; CAS: 60-00-4
EGTA	Sigma-Aldrich	E3889; CAS: 67-42-5
Sodium Chloride (NaCl)	Sigma-Aldrich	S7653; CAS: 7647-14-5
Sodium Dodecyl Sulfate (SDS)	Sigma-Aldrich	436143; CAS: 151-21-3
Trizma base	Sigma-Aldrich	93362; CAS: 77-86-1
Sodium deoxycholate	Sigma-Aldrich	D6750; CAS: 302-95-4
Nonidet P 40 Substitute	Sigma-Aldrich	74385; CAS: 9016-45-9
Methanol	Sigma-Aldrich	322415; CAS: 67-56-1
Ethanol	Sigma-Aldrich	51976; CAS: 64-17-5
Protease Inhibitor Cocktail (100X)	Cell Signaling Technology	5871
Fetal Bovine Serum (FBS)	GIBCO, Thermo Fisher Scientific	A31604-01
Human Serum -TypeAB male	Euroclone	ECS0219D
Dulbecco’s Modified Eagle medium (DMEM)	Sigma-Aldrich	D5671
RPMI-1640	Corning	15-040-CV
Iscove’s Modified Dulbecco’s Medium (IMDM)	Corning	10-016-CV
Penicillin-Streptomycin Mixture	Lonza	DE17-602E
L-Glutamine 200mM	Euroclone	ECB3000D
2-Mercaptoethanol (50mM) for cell culture	Thermo Fisher Scientific	31350010; CAS: 60-24-2
β-Estradiol	Sigma-Aldrich	E2758; CAS: 50-28-2
Trypsin/EDTA (10X)	Lonza	BE02-007E
Lymphoprep	StemCell Technologies	07851
TWEEN 20 non-ionic, aqueous solution, 10% (w/v)	Sigma-Aldrich	11332465001
Dithiothreitol (DTT)	Thermo Fisher Scientific	707265ML; CAS: 3483-12-3
Betaine solution	Sigma-Aldrich	B0300; CAS: 107-43-7
Magnesium Chloride 1M	Ambion	M1028; CAS: 7786- 30-3
RNase Inhibitor	Applied Biosystems, Thermo Fisher Scientific	N8080119
Glycerol	Sigma-Aldrich	G5516; CAS: 56-81-5
Ampicillin sodium salt	Sigma-Aldrich	A0166; CAS: 69-52-3
Polybrene	Sigma-Aldrich	107689
Puromycin dihydrochloride	Sigma-Aldrich	P8833; CAS: 58-58-2

**Critical commercial assays**		

Fast SYBR Green Master Mix	Thermo Fisher Scientific	4385618
ReliaPrep RNA Cell Miniprep System	Promega	Z6012
Buffer RLT	QIAGEN	79216
RNeasy Plus Micro kit	QIAGEN	74034
ImProm-II Reverse Transcription System	Promega	A3803
SuperScript IV VILO Master Mix	Thermo Fisher Scientific	11756050
CD14 MicroBeads	Miltenyi	130-050-201
GeneArt Precision gRNA synthesis kit	Thermo Fisher Scientific	A29377
P3 Primary Cell 4D-Nucleofector kit	Lonza	V4XP-3024
TOPO TA cloning kit for sequencing	Thermo Fisher Scientific	450030
Direct cAMP ELISA kit	Enzo Life Sciences	ADI-901-066
Mouse IL-10 DuoSet ELISA	R&D Systems	DY417
Prostaglandin E2 Express Elisa Kit	Cayman Chemical	CAY-500141
Bright-Glo Luciferase Assay System	Promega	E2650
QIAamp DNA Micro kit	QIAGEN	56304
Chromium Singe Cell 3′ Reagent kit v2	10X Genomics	PN-120237
Chromium i7 Multiplex Kit	10X Genomics	PN-120262
High Sensitivity D5000 ScreenTape	Agilent Technologies	5067-5592
High Sensitivity D5000 Reagents	Agilent Technologies	5067-5593
D1000 Reagents	Agilent Technologies	5067-5583
D1000 ScreenTape	Agilent Technologies	5067-5582
Loading Tips TapeStation System	Agilent Technologies	5067-5599
Qubit dsDNA HS Assay Kit	Thermo Fisher Scientific	Q32854
SuperScript II Reverse Transcriptase	Thermo Fisher Scientific	18064071
Nextera XT Index Kit (24 indexes, 96 samples)	Illumina	FC-131-1001
TruSeq ChIP Library Prep kit	Illumina	IP-202-1012
GoTaq Hot Start Polymerase	Promega	M5008
KAPA HiFi HotStart ReadyMix	Roche	07958935001
Nextera XT DNA library preparation kit (96 samples)	Illumina	FC-131-1096
One Shot Stbl3 Chemically Competent *E. coli*	Thermo Fisher Scientific	C737303

**Deposited data**		

Raw and analyzed RNA-Seq data	This paper	ArrayExpress: E-MATB-9275
Raw and analyzed scRNA-Seq data	This paper	ArrayExpress: E-MATB-9253
Raw and analyzed ChIP-Seq data	This paper	ArrayExpress: E-MATB-9254
Raw and analyzed ATAC-Seq data	This paper	ArrayExpress: E-MATB-9252

**Experimental models: cell lines**		

Hematopoietic progenitor cell line (Hoxb8-FL)	Hans Hacker	Redecke et al., 2013
L929, m-CSF producer	Ostuni et al., 2013	
SP2/0, Flt3-ligand producer	Federica Benvenuti	
ISRE_LUC	Francesca Granucci	Jiang et al., 2005

**Experimental models: organisms/strains**		

Mouse: C57BL/6NCrl	Charles River Laboratories	027C57BL/6
Mouse: VavCre C57BL/6	Michael Huber	N/A
Mouse: VavCre x Mef2c^−/−^Mef2d^fl/fl^ C57BL/6	Michael Huber	N/A
Mouse: MxCre x Stat3^fl/fl^ C57BL/6	Valeria Poli	N/A
Mouse: Rosa26-Cas9 Knockin	Luigi Naldini	Stock No: 024858

**Oligonucleotides**		

See Table S7	This paper	N/A

**Recombinant DNA**		

lentiGuide-Puro	Addgene	52963; RRID:Addgene_52963
pGL3-promoter Luciferase Reporter Vector	Promega	E1751

**Software and algorithms**		

Fiji ImageJ v.2.0.0-rc-69	NIH software	https://imagej.net/Fiji/Downloads
FlowJo Software v.10.6.0	LLC	https://www.flowjo.com
GraphPad PRISM v.8.4.2	GraphPad Software	https://www.graphpad.com
Image Lab Software	Biorad	https://www.bio-rad.com/
Agilent TapeStation Software	Agilent	https://www.agilent.com/
Integrative Genome Viewer (IGV) v.2.8.2	Broad Institute	http://software.broadinstitute.org
STAR aligner v.2.5.3	Dobin et al., 2013	https://github.com/alexdobin/STAR
BWA aligner v.0.7.15	Li and Durbin, 2009	http://bio-bwa.sourceforge.net/
Cell Ranger v2.0	Zheng et al., 2017	https://support.10xgenomics.com/single-cell-gene-expression/software/pipelines/latest/what-is-cell-ranger
SRA Toolkit v.2.8		https://github.com/ncbi/sra-tools
MACS2 v.2.2.1	Zhang et al., 2008	https://github.com/macs3-project/MACS
Samtools v1.4	Li et al., 2009	http://samtools.sourceforge.net/
BEDTools v.2.24.0	Quinlan and Hall, 2010	https://bedtools.readthedocs.io/en/latest/content/bedtools-suite.html
deepTools v2.4.0	Ramírez et al., 2014	https://deeptools.readthedocs.io/en/develop/
R v.3.4.1 or v3.5.2 (for Seurat)		https://www.r-project.org
ChromHMM v1.22	Ernst and Kellis, 2012	http://compbio.mit.edu/ChromHMM/
Rsubread v1.24.2	Liao et al., 2019	http://bioconductor.org/packages/release/bioc/html/Rsubread.html
edgeR v3.20.7	Robinson et al., 2010	http://bioconductor.org/packages/release/bioc/html/edgeR.html
Seurat v3.1.2		https://satijalab.org/seurat/
Monocle3 v.0.1.3	Trapnell et al., 2014	https://cole-trapnell-lab.github.io/monocle3/
ChIPpeakAnno v3.16.1	Zhu et al., 2010	https://www.bioconductor.org/packages/release/bioc/html/ChIPpeakAnno.html
ggplot2 v2.2.1		https://ggplot2.tidyverse.org/
ComplexHeatmap v1.6.0		https://bioconductor.org/packages/release/bioc/html/ComplexHeatmap.html
pheatmap v1.0.10		https://cran.r-project.org/web/packages/pheatmap/index.html
GSEA v.4.0.3	Subramanian et al., 2005	https://www.gsea-msigdb.org/gsea/index.jsp
HOMER v4.10	Heinz et al., 2010	http://homer.ucsd.edu/homer/motif/

### Resource availability

#### Lead contact

Further information and requests for resources and reagents should be directed to and will be fulfilled by the Lead Contact, Renato Ostuni (ostuni.renato@hsr.it).

#### Materials availability

This study did not generate new unique reagents.

#### Data and code availability

The accession numbers for the data reported in this paper are: ArrayExpress: E-MATB-9275 (bulk RNA-Seq), ArrayExpress: E-MATB-9253 (scRNA-Seq), ArrayExpress: E-MATB-9254 (ChIP-Seq), and ArrayExpress: E-MATB-9252 (ATAC-Seq).

### Experimental model and subject details

#### Animal used in this study

Animal experiments were performed in accordance with the Italian Laws (D.L.vo 116/92), which enforce the EU 86/609 Directive (approved by the Italian Ministry of Health, #449/2018-PR). C57BL/6 mice were purchased from Charles River Italy. Rosa26-Cas9 genetically targeted mice (MGI, J:213550) were obtained from Luigi Naldini. Bone marrow cells from MEF2C-D double deficient (*Mef2d*^*−/−*^ *Mef2c*^*fl/fl*^ x *Vav-Cre*) or MEF2C- and MEF2D-proficient (*Vav-Cre*) mice were provided by Michael Huber (University Clinic Aachen). Bone marrow cells from STAT3-deficient (*Stat3*^*fl/fl*^ *Mx1-Cre*) or STAT3-proficient (*Stat3*^*fl/fl*^) mice were provided by Valeria Poli (University of Turin).

### Method details

#### Differentiation and culture of murine cells

Bone marrow cells were collected from femurs and tibias in 50 mL PBS, filtered through a 70 μm cell strainer and centrifuged 450 x g for 5 minutes. Red blood cells were lysed using 0.2% NaCl solution, followed by 1.6% NaCl solution. Cells were filtered through a 70 μm cell strainer and centrifuged 450 x g for 5 minutes. For BMDM differentiation, 5 × 10^4^ – 5 × 10^6^ bone marrow cells were plated and cultured in IMDM supplemented with 10% FBS, 20% L929-conditioned medium containing M-CSF, antibiotics (penicillin G 100 U/ml and streptomycin sulfate 100 U/ml), 2 mM L-glutamine and 5 μM 2-mercaptoethanol. After six days of culture, adherent cells were > 99% Cd11b^+^ F4/80^+^, as assessed by flow cytometry, and were stimulated as described below. For BMDC differentiation, 1.5 × 10^6^ bone marrow cells were plated and cultured in IMDM supplemented with 10% FBS, 5 μM 2-mercaptoethanol, antibiotics (penicillin G 100 U/ml and streptomycin sulfate 100 U/ml), 2 mM L-glutamine and 15% of FLT3 ligand-containing supernatant, produced from an SP2/0 transfected cell line that secretes murine recombinant FLT3 ligand. To achieve BMDC differentiation, medium was replaced every three days. At day 7 of culture cells were harvested, plated at 2 × 10^5^ cells/100 μl in 96-well U-bottom plates as previously described (Caronni et al., 2018).

Conditionally immortalized Hoxb8-FL cells (Redecke et al., 2013) were cultured in RPMI-1640 supplemented with 10% FBS, 5% of FLT3 ligand-containing supernatant, antibiotics (penicillin G 100 U/ml and streptomycin sulfate 100 U/ml), 2 mM L-glutamine, 5 μM 2-mercaptoethanol and 1 μM β-estradiol. For iMac differentiation, Hoxb8-FL cells were washed twice with PBS and plated in IMDM supplemented with 10% FBS, 20% L929-conditioned medium containing M-CSF, antibiotics (penicillin G 100 U/ml and streptomycin sulfate 100 U/ml), 2 mM L-glutamine and 5 μM 2-mercaptoethanol. At day 5 of culture, fresh L929-conditioned medium was added. After 7 days of culture, adherent cells were > 99% Cd11b^+^ F4/80^+^, as assessed by flow cytometry, and were stimulated as indicated below.

Peritoneal macrophages were obtained by flushing the peritoneal cavity with 5 mL of cold PBS supplemented with 2% of Fetal Bovine Serum (FBS) and centrifuged 400 x g for 5 minutes. Cells were cultured in Iscove’s Modified Dulbecco’s Medium (IMDM) supplemented with 10% FBS, 20% L929-conditioned medium containing M-CSF, antibiotics (penicillin G 100 U/ml and streptomycin sulfate 100 U/ml), 2 mM L-glutamine and 5 μM 2-mercaptoethanol. After 24 hours, cells were washed twice with PBS to remove non-adherent cells and macrophages stimulated as described below.

#### Generation and culture of gene-edited cells

Single guide RNAs (sgRNAs) were designed using CHOPCHOP (Labun et al., 2019), and generated by *in vitro* transcription using GeneArt Precision gRNA Synthesis kit following manufacturer’s instructions. Ribonucleoprotein (RNP) complexes of Cas9-sgRNAs were obtained by incubating 30 μg or 5 μg of Cas9 (produced in-house or commercial, respectively) with 12 μg or 6 μg sgRNA for 15 minutes at room temperature. Hoxb8-FL cells (2.5 × 10^5^) or BMDMs (5 × 10^5^, day 4 of differentiation) were resuspended in P3 solution of P3 Primary Cell 4D-Nucleofector kit, mixed with RNP complex and electroporated using ED-113 program of the 4D-Nucleofector System (Lonza). BMDMs were washed 24 hours after nucleofection and stimulated after additional 24 hours. Hoxb8-FL cells were FAC-sorted as single clones in 96-well U-bottom plates (FACSAria II, BD Biosciences) 5 days after nucleofection and expanded in culture.

Gene-editing efficiency and clone screening were assessed via Non-Homologous End Joining (NHEJ) at targeted sites. Briefly, genomic DNA was purified using QIAamp DNA Micro kit and targeted regions were amplified by PCR. PCR products were purified with AMPure XP beads, quantified by NanoDrop 8000 and mixed 1:1 with PCR product from wild-type cells. Annealed PCR products (500 ng) were digested with T7 Endonuclease for 30 minutes at 37°C and subjected to capillary electrophoresis using D1000 TapeStation kit (Agilent 4200 TapeStation). NHEJ efficiency was defined by calculating the percentage of cleavage of the PCR product. Gene-edited Hoxb8-FL clones were validated by Sanger Sequencing using TOPO-TA Cloning Kit following manufacturer’s instructions. When the editing occurs within coding sequence, protein disruption was validated by Western Blot analyses either in BMDMs or in Hoxb8 clones upon differentiation in iMacs.

#### Differentiation of human monocyte derived macrophages (hMDMs)

Human peripheral blood leukocyte concentrates from healthy donors were obtained in accordance with the Declaration of Helsinki and with Ospedale San Raffaele ethics committee approval (TIGET09 protocol). Peripheral blood mononuclear cells (PBMCs) were isolated by density centrifugation over Lymphoprep gradient. CD14^+^ cells were obtained from PBMC by positive selection with CD14 MicroBeads according to the manufacturer’s instructions. 3 × 10^6^ CD14^+^ cells were plated in 6-well plate in Dulbecco’s Modified Eagle medium (DMEM) supplemented with 10% FBS, 5% human type AB serum male, antibiotics (penicillin G 100 U/ml and streptomycin sulfate 100 U/ml) and 2mM L-glutamine. At day 7 of culture, macrophage differentiation was assessed by morphological analyses.

#### *Ex vivo* stimulation of mouse and human cells

Cells were stimulated with reagents at the following concentrations: LPS (10 ng/ml for mouse and human macrophages; 1 μg/ml for BMDCs and for IFN-β quantification in BMDMs or iMacs), PGE_2_ (1 μM), IL-10 (10 ng/ml), IL-4 (10 ng/ml), IFN-α (10 U/ml), IFN-β (100U/ml), DMXAA (10 μg/ml), poly(I:C) (10 μg/ml), CpG (100 nM), PFI-1 (10 μM, 2-hour pre-treatment), SGC-CBP30 (10 μM, 2-hour pre-treatment), forskolin (50 μM, 2-hour pre-treatment), 6-Bnz-cAMP (250 μM, 2-hour pre-treatment), db-cAMP (50 μM, 2-hour pre-treatment), salmeterol xinafoate (2.5 μM), BAY60-6583 (10 μM), anti-IL10-R antibody or isotype control (10μg/ml, 40-minute pre-treatment).

#### *In vivo* stimulation of peritoneal macrophages or bronchoalveolar lavage fluid (BALF) cells

C57BL/6 mice were intraperitoneally injected with PGE_2_ (2 μg per mouse) or an equal volume of PBS. After 1 hour, mice were intraperitoneally injected with LPS (10 μg per mouse) given alone or in combination with PGE_2_ (2 μg per mouse). Mice were sacrificed 2 hours post injection and peritoneal lavage was collected as described above. Peritoneal macrophages (CD11b+ F4/80+) were FAC-sorted in RLT buffer (QIAGEN) on a FACSAria II instrument (BD Biosciences). Total RNA was extracted as described below.

For the stimulation of BALF cells, C57BL/6 mice were anesthetized and intranasally administered PGE_2_ (2 μg per mouse) or an equal volume of PBS. After 1 hour, mice were intranasally administered LPS (10 μg per mouse) alone or in combination with PGE_2_ (2 μg per mouse). Mice were sacrificed 2 hours post treatment and BALF cells collected in 3 mL of cold PBS. To assess the percentage of alveolar macrophages, cells were stained with Cd11c (1:100) and Siglec F (1:100). Total RNA was extracted as described below. Details of antibodies used are reported in the Key resources table.

#### Flow cytometry

To assess macrophages differentiation, cells were washed with cold PBS and incubated for 30 minutes at 4°C with conjugated antibodies at the indicated concentrations: F4/80 (1:100), Cd11b (1:100), Cd11c (1:100), Ly6c (1:100). To assess cell apoptosis and viability, cells were washed with cold PBS and resuspended in AnnexinV binding buffer (PE AnnexinV Apoptosis Detection kit). Cells were stained following manufacturer’s instructions. Details of antibodies and kit used are reported in the Key Resource Table. Cells were washed, resuspended in PBS-BSA 1% and analyzed on a FACSCanto II (BD Bioscience). Data were analyzed with FlowJo Software 10.6.0.

#### Production of lentiviral vectors and transduction of BMDMs

Single gRNAs targeting *Hdac5* gene were designed using tools available from the Genetic Perturbation Platform (Broad Institute) and cloned into lentiGuide-Puro plasmid. Lentiviral vectors (LV) were obtained transfecting 293T cells with a solution containing a mix of the selected LV genome transfer plasmid, the packaging plasmids pMDLg/pRRE and pCMV.REV, pMD2.G and pAdvantage, as previously described (Milani et al., 2019). Medium was changed 14 to 16 hours after transfection and supernatant collected 30 hours after medium change. Vector-containing supernatants were passed through a 0.22-μm filter, transferred into sterile polyallomer tubes and centrifuged at 20,000 x g for 120 min at 20°C (Beckman Optima XL-100 K Ultracentrifuge). LV pellet was dissolved in the appropriate volume of PBS to allow 500 × to 1000 × concentrations. 2.5 × 10^5^/well Cas9-expressing BMDMs were transduced twice at day 5 and 6 of differentiation with a multiplicity of infection (MOI) of 10 in L929-conditioned medium supplemented with polybrene (8 μg/ml). After the second hit, transduced cells were selected with puromycin (5 μg/ml) for 48 hours and then stimulated as indicated.

#### Luciferase reporter assay

Selected genomic regions (Table S7) were cloned in the pGL3-promoter luciferase reporter vector using NheI and XhoI restriction enzymes. 5x10^5^ BMDMs (at day 4 of differentiation) were resuspended in P3 solution of P3 Primary Cell 4D-Nucleofector kit and electroporated with 1μg of the reporter. Electroporated BMDMs were plated at 1x10^5^ cells/well in 96-well plates, medium was replaced after 24 hours and cells stimulated after additional 24 hours. Luminescence was measured using Bright-Glo Luciferase Assay System, following manufacturer’s instructions.

For the +7.25 kb *Ifnb1* enhancer, site direct mutagenesis was achieved by PCR amplification of plasmid template with primers introducing the substitution of the MEF2A binding site (CCCAAAATAG) with the nucleotides GAGCTAGCGA, harboring an additional NheI restriction site. PCR products were transformed in Stbl3 bacteria. Positive clones were screened via NheI digestion of the plasmid obtained.

#### Immunofluorescence

BMDMs or iMacs were seeded in a 6-well plate at a density of 3.5 × 10^5^ cells/well and differentiated for 5 days as described above. Cells were then detached with trypsin and plated on coverslips within a 6-well plate at a density of 10^6^ cells/well. After 24 hours, cells were stimulated as indicated, washed with PBS and fixed in 1% paraformaldehyde (PFA) in PBS at room temperature for 10 minutes. Fixed cells were then permeabilized with cold methanol 100% for 10 minutes at −20°C, washed three times with 0.3% Triton X-100 in PBS, and blocked for 1 h with 5% BSA in 0.3% Triton X-100/PBS. Then, cells were stained with anti-NFκB (1:100) or anti-phospho TBK1 (1:50) overnight at 4°C. Cells were incubated with Alexa Fluor 488 Goat anti-Rabbit antibody (1:2,000), counterstained with DAPI (1:10,000) for 10 minutes at room temperature and mounted with Aqua/Poly mount on slides. Images were taken on a Nikon Eclipse E600 microscope (1024 × 1024, 40hex) and analyzed with Fiji ImageJ software (v 2.0.0-rc-69). Details of antibodies used are reported in the Key Resource Table.

#### TLR4 internalization assay

2 × 10^5^ BMDMs or iMacs were subjected to the indicated treatments and then washed with cold PBS. Cells were stained with anti-TLR4 antibody (1:200) as previously described (Zanoni et al., 2011) and LYVEDEAD Fixable Yellow (1:1,000) for dead-cell exclusion for 20 minutes on ice and then washed with cold PBS. Cells were resuspended in PBS and analyzed on FACSCanto II (BD Bioscience). Data were analyzed with FlowJo Software 10.6.0.

#### Western Blot analyses

5 × 10^6^ BMDMs or iMacs were subjected to the indicated treatments and lysed with a buffer containing 10 mM Tris-HCl pH 8, 1 mM EDTA pH 8, 140 mM NaCl, 1% Triton X-100, 0.1% SDS, 0.1% deoxycholate and protease/phosphatase inhibitors. Lysates were electrophoresed and immunoblotted with the following antibodies: anti-β-ACTIN (1:2,000), anti-VINCULIN (1:1,000), anti-IκBα (1:1,000), anti-p38 MAPK (1:1,000), anti-phospho p38 MAPK (Thr180/Tyr 182, 1:1,000), anti-SAPK/JNK (1:1,000), anti-phospho SAPK/JNK (Thr183/Tyr185, 1:1,000), anti-IRF1 (1:1,000), anti-phospho STAT1 (Tyr 701, 1:1,000), anti-phospho STAT2 (Tyr689, 1:100), anti-CREB (1:1,000), anti-phospho CREB (Ser133, 1:500), anti-HDAC5 (1:500), anti ERK5 (1:1,000), anti-phospho ERK5 (Thr218/Tyr220, 1:500).

To analyze nuclear translocation of IRF3, 10^7^ BMDMs were lysed with a buffer containing 50 mM HEPES, 1 mM EDTA pH 8, 140 mM NaCl, 0.25% Triton X-100, 0.5% NP-40, 10% glycerol and protease/phosphatase inhibitors. Lysates were incubated 10 minutes in ice and centrifuged 450 x g for 5 minutes. Supernatant was collected as cytosolic fraction and nuclei were washed with a buffer containing 10 mM Tris-HCl pH 8, 1 mM EDTA pH 8, 200 mM NaCl, 0.5 mM EGTA and protease/phosphatase inhibitors, incubated 10 minutes at room temperature on rotation and centrifuged 450 x g for 5 minutes. Nuclei were lysed with a buffer containing 10 mM Tris-HCl pH 8, 1 mM EDTA pH 8, 140 mM NaCl, 1% Triton X-100, 0.1% SDS, 0.1% deoxycholate and protease/phosphatase inhibitors. Lysates were electrophoresed and immunoblotted with the following antibodies: anti-IRF3 (1:1,000), anti-LAMINB1 (1:500).

#### Real-Time Quantitative PCR

If not differently stated, total RNA was extracted using ReliaPrep RNA Cell Miniprep System and quantified using NanoDrop 8000. Single-stranded cDNA was synthesized using ImProm-II Reverse Transcription System starting from 500 ng total RNA. For *in vivo* stimulated peritoneal macrophages, total RNA was extracted by RNeasy plus micro kit, following manufacturer’s instructions. Single-stranded cDNA was synthesized using SuperScript IV Vilo Master Mix. Amplification of target genes was performed using Fast SYBR Green Master Mix on a ViiA 7 Real-Time PCR System. Details of primer pairs used are reported in Table S7 and in the Key Resource Table.

#### Quantification of IFN-β

Cells were stimulated as indicated for 24 hours and supernatant was collected and centrifuged to remove cellular debris. L929 cells transfected with an interferon-sensitive luciferase construct (ISRE-Luc) (Jiang et al., 2005) were plated at 4 × 10^4^ cells/well in 96-well plate and after 8 hours were incubated over-night with conditioned supernatant and luminescence was measured using Bright-Glo Luciferase Assay System. Recombinant IFN-β was used for standard curve calibration.

#### Quantification of PGE_2_, IL-10, cyclic AMP

For the quantification of PGE_2_ or IL-10 release, cells were stimulated for the indicated time and supernatant was collected and centrifuged to remove cellular debris. IL-10 (Mouse IL-10 DuoSet ELISA kit) or PGE_2_ (Prostaglandin E_2_ Express ELISA Kit) were measured following manufacturer’s instructions. For the quantification of intracellular cyclic AMP, cells were stimulated as indicated and lysed in 0.1 M HCl. cAMP was quantified using Direct cAMP ELISA kit following manufacturer’s instructions. Absorbance was measured on a Multiskan GO Microplate Spectrophotometer.

#### Bacterial infections and CFU assay

3 × 10^5^ macrophages/well were infected in L929-conditioned medium without antibiotics at MOI 1 with *M. tuberculosis* H37Rv NCTC7416 or *M. bovis* BCG (OncoTICE MSD). Bacterial stocks were pre-quantified for colony forming unit (CFU) values and maintained at −80°C in Middlebrook 7H9, 10% OADC, 0.05% Tween80 supplemented with 20% glycerol. Upon thawing, bacterial stocks were centrifuged 17,000x g for 5 minutes, washed with PBS, resuspended in L929-conditioned medium without antibiotics, de-clumped by 10 passages through a 21-gauge needle and diluted in L929-conditioned medium immediately before the infection. Four hours or one day after infection, macrophages were lysed for RNA analyses or supernatant collected for IFN-β quantification, respectively.

#### VSV infection

5 × 10^5^ iMacs were infected with Vesicular Stomatitis Virus (VSV) at MOI of 20 or 2 in DMEM without serum for 1 hour at 37°C and then were supplemented with L929-conditioned medium. Three hours or one day after infection, macrophages were lysed for RNA analyses or supernatant collected for IFN-β quantification, respectively.

#### Generation and processing of bulk RNA-Seq data

##### RNA-Seq data generation

Total RNA was purified using the ReliaPrep RNA Cell Miniprep System and RNA-Seq libraries were generated using the Smart-seq2 method (Picelli et al., 2014). Five ng of RNA were retrotranscribed, cDNA was PCR-amplified (15 cycles) and purified with AMPure XP beads. After purification, the concentration was determined using Qubit 3.0 and size distribution was assessed using Agilent 4200 TapeStation system. Then, the tagmentation reaction was performed starting from 0.5 ng of cDNA for 30 minutes at 55°C and the enrichment PCR was carried out using 12 cycles. Libraries were then purified with AMPure XP beads, quantified using Qubit 3.0, assessed for fragment size distribution on an Agilent 4200 TapeStation system. Sequencing was performed on an Illumina NextSeq500 or NovaSeq6000 (single-end, 75bp read length) following manufacturer’s instruction.

##### RNA-Seq data processing

Reads were aligned to the mm10 reference genome using STAR aligner (v 2.5.3) (Dobin et al., 2013); the featureCounts function from Rsubread package (v 1.24.2) (Liao et al., 2019) was used to compute reads over RefSeq *Mus musculus* transcriptome (mm10) (Pruitt et al., 2007), setting minMQS option to 255. Further analyses were performed in R environment (v 3.4.1) with edgeR R package (v. 3.20.7) (Robinson et al., 2010). Read counts of expressed genes were normalized with the Trimmed Mean of M-values (TMM) method (Robinson and Oshlack, 2010) using calcNormFactors function. Dispersion was estimated with the estimateDisp function. Differential expression across different conditions was evaluated fitting a negative binomial generalized linear model on the dataset with glmQLFit function and then performing a quasi-likelihood (QL) F-test with glmQLFTest function. Sample replicates were included in the design as covariates. Reads per kilo base per million (RPKM) values were computed for each gene with rpkm function. For published RNA-Seq datasets, fastq files were downloaded from GEO repository using fastq-dump from SRA Toolkit (v. 2.8) and processed as described above.

#### RNA-Seq analyses of costimulated BMDMs

##### Definition of stimulus-inducible genes

After RNA-Seq data processing, as described above, genes with RPKM > 1 in at least two samples in the datasets were retained. We defined induced genes by comparing their expression in the LPS, IFN-α, PGE_2_, IL-10 or IL-4 conditions setting log_2_FC(RPKM) ≥ 2 and FDR < 0.05 versus untreated (UT) controls as cut-offs. After filtering out genes not reaching RPKM > 1.5 in at least two replicates within each comparison, we obtained 468 LPS-induced, 291 IFN-α-induced, 138 PGE_2_-induced, 28 IL-10-induced and 118 IL-4-induced genes.

##### Definition of induced genes sensitive or resistant to costimulation

For the analysis of LPS-stimulated transcription, we filtered out genes induced by PGE_2_, IL-10 or IL-4, leading to the definition of a final set of 421 LPS-inducible genes. These were classified as ‘PGE_2_-sensitive’ if having log_2_FC(RPKM) ≤ −2 and FDR < 0.05 in the LPS+PGE_2_ versus LPS condition (n = 70); using the same analytical strategy and cut-offs, we defined ‘IL-10-sensitive’ (n = 72) and ‘IL-4-sensitive’ genes (n = 42). Genes were classified as ‘resistant’ (n = 72) if their expression was preserved in all costimulation conditions (LPS+PGE_2_, LPS+IL-10, LPS+IL-4) as compared to LPS alone, setting a cut-off of −1 < log_2_FC(RPKM_LPS+costim_/RPKM_LPS_) < 1. For the analysis of IFN-α-stimulated transcription, we focused on 283 genes that were induced by IFN-α but not by PGE_2_ and IL-10. We classified 5 genes as PGE_2_-sensitive and 0 as IL-10 sensitive [(log_2_FC(RPKM_costim_ /RPKM_IFN-α_) ≤ −2 and FDR < 0.05] and 251 genes as resistant [-1 < log_2_FC(RPKM_costim_ /RPKM_IFN-α_) < 1].

#### Nascent RNA-Seq analyses of lipid A-stimulated BMDMs

##### Definition of MYD88-, TRIF-, IRF3- or IFNAR-dependent genes

We downloaded and processed RNA-Seq datasets (GSE67357) from wt or gene-deficient BMDMs stimulated with lipid A for 120 minutes (WT0, WT0 Rep2, WT120, WT120 Rep2, MYD88-120, MYD88-120 Rep2, TRIF-120, TRIF-120 Rep2, IRF3-120, IRF3-120 Rep2, IFNAR-120, IFNAR-120 Rep2) (Tong et al., 2016). Genes with RPKM > 1 in at least two samples in the datasets were retained for further analyses. For each expressed gene and for each experimental condition we computed mean expression (RPKM) across replicates. For each of the previously defined set of 421 LPS-induced genes, we calculated the percentage of expression in *MyD88*^*−/−*^, *Ticam1*^*−/−*^, *Irf3*^*−/−*^ or *Ifnar*^*−/−*^ versus wt BMDMs stimulated with lipid A (RPKM_KO_lipidA_^∗^100/RPKM_WT_lipidA_). We set the percentage of expression to 100 for those LPS-induced genes that were not expressed in this dataset (we assumed no difference between the considered conditions). Genes whose expression in genetically ablated versus wt BMDMs was below 30% were classified as MYD88-, TRIF-, IRF3- or IFNAR-dependent, respectively.

##### Gene set enrichment analysis (GSEA)

For each genotype, expressed genes were ranked by decreasing order of log_2_FC(RPKM) in lipid A-stimulated genetically ablated versus wt BMDMs. GSEA (v. 4.0.3) (Subramanian et al., 2005) was performed on ranked gene lists using previously defined PGE_2_-sensitive and resistant transcripts as Gene Sets, with number of permutations set to 10,000.

#### RNA-Seq analyses of BMDMs treated with exogenous IFN-β

##### Definition of IFNβ-restored genes

After RNA-Seq data processing, genes with RPKM > 1 in at least two samples in the datasets were retained. Previously defined PGE_2_-sensitive genes were classified as ‘restored’ (n = 33) if having log_2_FC(RPKM) ≥ 1 in the LPS+PGE_2_+IFN-β versus LPS+PGE_2_ condition. Genes with 0,5 ≤ log_2_FC(RPKM_LPS+PGE2+IFNb_/RPKM_LPS+PGE2_) < 1 were classified as ‘partially restored’ and genes with log_2_FC(RPKM_LPS+PGE2+IFNb_/RPKM_LPS+PGE2_) < 0,5 were classified as ‘not restored’. We calculated also a percentage of restoration in LPS+PGE_2_+IFN-β versus LPS+PGE_2_ treated BMDMs [(RPKM_LPS+PGE2+IFNb_ - RPKM_LPS+PGE2_)/(RPKM_LPS_ - RPKM_LPS+PGE2_)].We set the percentage of restoration to 0 for those LPS-induced genes that were not expressed in this dataset (we assumed no difference between the considered conditions).

#### RNA-Seq analyses of BMDMs treated with IL-10R blocking antibody

##### Definition of anti-IL-10R-restored genes

After RNA-Seq data processing, genes with RPKM > 1 in at least two samples in the datasets were retained. We calculated a percentage of restoration for each of the previously defined PGE_2_-sensitive genes [(RPKM_LPS+PGE2+aIL-10R_ - RPKM_LPS+PGE2_)/(RPKM_LPS_ - RPKM_LPS+PGE2_)]. Genes were classified as ‘restored’ (n = 9) if having percentage of restoration ≥ 0,8 and ‘partially restored’ if having 0,2 ≤ percentage of restoration < 0,8. Genes with percentage of restoration < 0,2 were classified as ‘not restored’.

#### RNA-Seq analyses of LPS-stimulated BMDMs with BRD2-4 or CBP-p300 inhibitors

RNA-Seq data were generated and pre-processed as described above. Genes not passing the expression cut-off of RPKM > 1 in at least three samples in the datasets were filtered out. For each gene and for each experimental condition we computed mean expression (RPKM) across replicates. For each of the previously defined set of 421 LPS-induced genes, we calculated the percentage of expression in BMDMs stimulated with LPS in the presence or absence of BRD2-4 or CBP-p300 inhibitors (RPKM_inhibitor_LPS_^∗^100/RPKM_Ctrl_LPS_). We set the percentage of expression to 100 for those LPS-induced genes that were not expressed in this dataset. Genes whose expression was below 30% in LPS-stimulated BMDMs treated with inhibitors versus controls were classified as BRD2-4 or CBP-p300 dependent, respectively.

#### RNA-Seq analyses of wt or MEF2A-deficient iMacs

##### Definition of LPS-inducible genes in wt iMacs

RNA-Seq data were generated and pre-processed as described above. Genes not passing the expression cut-off of RPKM > 1 in at least one sample in the dataset were filtered out. Differential gene expression was performed considering MEF2A-deficient iMac clones (D7, A7, A8, C7) and MEF2A-proficient (referred to as wt) iMac clones (NE, B3 and D10) as sets of biological replicates. We defined LPS-inducible genes in wt iMacs by comparing their expression in the LPS versus UT conditions, setting log_2_FC(RPKM) ≥ 2 and FDR < 0.05 as cut-offs. After filtering out genes not reaching RPKM > 1.5 in two samples within each comparison, and genes that did not pass the cut-off for induction by LPS also in wt BMDMs, we defined a set of 312 LPS-inducible genes in iMacs.

##### Definition of MEF2A-dependent or MEF2A-independent genes in LPS-treated iMacs

We compared expression of LPS-inducible genes in MEF2A-deficient versus wt iMacs upon LPS-stimulation. Of the previously defined set of 312 LPS-inducible in iMacs, 94 were classified as MEF2A-dependent, setting log_2_FC(RPKM_MEF2Ako_LPS_/RPKM_WT_LPS_) ≤ −2 and FDR < 0.05 as cut-offs. As a control group of MEF2A-independent transcripts, we selected 118 genes whose expression in response to LPS was not affected in MEF2A-deficient as compared to wt iMacs, setting −1 < log_2_FC(RPKM_MEF2Ako_LPS_/RPKM_WT_LPS_) < 1 as cut-offs.

##### Overlap between PGE_2_-sensitive or resistant and MEF2A-dependent or MEF2A-independent genes

Of the previously defined sets of PGE_2_-sensitive or resistant genes in BMDMs, only those that were induced by LPS in iMacs were retained (50/70 and 50/72 genes, respectively) for these analyses. We calculated the overlap between these sets of genes and those, previously defined, of MEF2A-dependent or MEF2A-independent genes in iMacs, an hypergeometric tests was performed in order to assess significance of these overlaps. Out of 50 PGE_2_-sensitive genes (induced by LPS in both BMDMs and iMacs), 22 (44%) were MEF2A-dependent and 14 (28%) were MEF2A-independent. Conversely, out of 50 resistant genes (induced by LPS in both BMDMs and iMacs) 1 (2%) was MEF2A-dependent and 34 (68%) were MEF2A-independent.

##### Gene set enrichment analysis

For each genotype, expressed genes were ranked by decreasing order of log_2_FC(RPKM_MEF2Ako_LPS_/RPKM_WT_LPS_) in LPS-stimulated MEF2A-deficient versus wt iMacs. GSEA (v. 4.0.3) (Subramanian et al., 2005) was performed on ranked gene lists using PGE_2_-sensitive (n = 70) and resistant (n = 72) transcripts as Gene Sets, with number of permutations set to 10,000.

#### RNA-Seq analyses of wt and MEF2C-D double-deficient BMDMs

RNA-Seq data were generated and pre-processed as described above. Genes not passing the expression cut-off of RPKM > 1 in at least three samples in the dataset were filtered out. Definition of LPS-induced genes was performed as described above. RPKM values were computed, as described above, and reported for previously defined PGE_2_-sensitive and resistant genes.

#### Generation and analysis of single-cell RNA-Seq data

##### Data generation

BMDMs were stimulated for 4 hours as indicated above, and scRNA-Seq libraries were generated using a microfluidics-based approach on Chromium Single-Cell Controller (10X Genomics) using the Chromium Single Cell 3′ Reagent Kit v2 according to the manufacturers’ instructions. Briefly, single cells were partitioned in Gel Beads in Emulsion (GEMs) and lysed, followed by RNA barcoding, reverse transcription and PCR amplification (12 cycles). The concentration of the scRNA-seq libraries was determined using Qubit 3.0 and size distribution was assessed using an Agilent 4200 TapeStation system. Libraries were sequenced on an Illumina NextSeq500 instrument (paired-end, 150bp read length).

##### Data processing

Fastq files were processed with Cell Ranger (v 2.0) (Zheng et al., 2017) using default parameters. Reads were aligned to reference genome mm10 and quantified using ENSEMBL genes (Hubbard et al., 2002) as gene model. Only confidently mapped reads, non-PCR duplicates, with valid barcodes and unique molecular identifiers (UMIs) were retained to compute a gene expression matrix containing the number of UMI for every cell and gene. Gene counts were imported in R environment (v. 3.5.2) and processed with Seurat (v 3.1.2) (https://satijalab.org/seurat/). Cells expressing less than 1000 unique genes were discarded. Genes with a mean expression (counts normalized with NormalizeData function) lower than 0.01 were excluded. Cells with a ratio of mitochondrial versus endogenous genes expression exceeding 0.1 were also excluded, resulting in 3,935 retained cells. Raw expression data were then scaled using SCTransform function, regressing on percentage of mitochondrial gene expression and cell cycle scores. Cell cycle scores were calculated using CellCycleScoring function.

##### Graph-based clustering and differential gene expression analyses

Shared Nearest Neighbor (SNN) graph was constructed using the FindNeighbors function taking as input the first 30 principal components, computed with RunPCA function. Cell clusters were defined using a resolution r = 0.4, calculated with the FindCluster function and were visualized in 2 dimensions using uniform manifold approximation and projection (UMAP) (Becht et al., 2018). Cluster-specific genes were identified using FindMarkers function with option only.pos = TRUE, setting a cut-off of FDR < 0.05. Additional, separate graph-based clustering analyses were performed on filtered sub-datasets including cells from the UT, PGE_2_, LPS and LPS+PGE_2_ samples (3,102 cells, sub-dataset A) as well as the UT, PGE_2_, IFN-α and IFN-α+PGE_2_ samples (2,162 cells, sub-dataset B). For each cell within sub-dataset A we computed the mean expression of LPS-induced or PGE_2_-induced genes, as well as PGE_2_-sensitive or resistant genes previously defined by bulk RNA-Seq analyses. For each cell within sub-dataset B we computed the mean expression of IFN-α-induced or PGE_2_-induced genes. Only genes expressed in the single cell dataset were considered.

##### Single-cell trajectories

The dataset (genes and cells filtered as described above) was then reanalyzed with Monocle3 (v. 0.1.3) (Trapnell et al., 2014) (https://cole-trapnell-lab.github.io/monocle3/). Data were normalized and principal component analysis was performed with preprocess_cds function with num_dim parameter set to 30. Dimensionality reduction was performed with reduce_dimension function using UMAP. Finally, trajectories were constructed using cluster_cells function and learn_graph function with use_partition parameter set to FALSE.

#### Generation and processing of ChIP-Seq data

##### ChIP-Seq data generation

Cells were stimulated for 4 hours (H3K27ac, IRF1, STAT1 ChIP-Seq) or 2 hours (PU.1, MEF2A, MEF2D, JUNB, NF-κB p65 ChIP-Seq). 6 × 10^6^ (H3K27ac or PU.1 ChIP-Seq) or 10^8^ (MEF2A, MEF2D, IRF1, STAT1, JUNB, NF-κB p65 ChIP-Seq) cells were fixed with 1% formaldehyde, and nuclear fractions isolated and lysed as described previously (Ostuni et al., 2013). Fragmented chromatin was obtained using Covaris E220 focused-ultrasonicator and nuclear extracts were incubated overnight at 4°C with Dynabeads Protein G, previously coupled with 3 μg (for H3K27ac), 5 μg (for PU.1) or 15 μg (for MEF2A, MEF2D, IRF1, STAT1, JUNB, NF-κB p65) of antibody. Details of antibodies used are reported in STAR Methods. Beads were recovered using a 96-well magnet, washed, and DNA de-crosslinked overnight at 65°C. DNA was purified with AMPure XP beads and quantified with Qubit 3.0. ChIP or input DNA (1 μl) were used for ChIP-qPCR experiments. ChIP DNA (5 ng) was used for library preparation with Illumina TruSeq ChIP Library Prep kit and sequenced on Illumina NextSeq500 or NovaSeq6000 (single-end, 75bp read length).

##### ChIP-Seq data processing, peak calling and normalization

Reads from fastq files were aligned to the mm10 reference genome using BWA aligner (v. 0.7.15) (Li and Durbin, 2009). Bam files were processed using samtools (v.1.4) (Li et al., 2009) and BEDTools (v. 2.24.0) (Quinlan and Hall, 2010) suites. Reads with a mapping quality lower than 15 or duplicated reads were discarded. For published ChIP-Seq datasets, fastq files were downloaded from GEO repository using fastq-dump from SRA Toolkit (v. 2.8) and processed as described above. All regions of interest were annotated using ChIPpeakAnno R package (v 3.16.1) (Zhu et al., 2010) and defined as proximal or distal if located within or beyond 5kb from an annotated TSS, respectively. Distal regions were further classified as intragenic or intergenic. Read counts were computed on all regions of interest using multiBamCov function from BEDTools. Further analyses were performed in R environment (v 3.4.1) with edgeR package (v. 3.20.7).

For H3K27ac ChIP-Seq, peak calling was performed using MACS2 (v. 2.2.1) (Zhang et al., 2008) callpeak function with parameters -g mm -q 0.01–broad–nomodel–extsize 147. Peaks with a q-value lower than 1e-3 were selected and resulting peaks with a distance lower than 1,000bp were merged using mergeBed function form BEDTools. Resulting peaks from all samples were then merged. Read counts were computed on resulting regions as described above and normalized with the TMM method using calcNormFactors function. Dispersion was estimated with the estimateDisp function. Differences in signal intensities across different conditions were evaluated fitting a negative binomial generalized linear model on the dataset with glmQLFit function and then performing a quasi-likelihood (QL) F-test with glmQLFTest function. Sample replicates were included in the design as covariates. CPM (counts per million) values were computed for each region with cpm function.

For PU.1, MEF2A, MEF2D, IRF1, STAT1, JUNB or NF-κB p65 ChIP-Seq, peak calling was performed using MACS2 (v. 2.2.1) (Zhang et al., 2008) callpeak function with parameters -g mm. For each sample, peaks with a q-value lower than 1e-5 were selected. Read counts were computed on regions of interest as described above. For PU.1, MEF2A and MEF2D ChIP-Seq data, CPM values were estimated by normalizing counts on total reads mapping within merged peaks for each TF. For the other transcription factors, which are LPS-inducible and thus displayed limited binding in untreated samples, counts were normalized by total library size.

#### Generation and processing of ATAC-Seq data

##### ATAC-Seq data generation

ATAC-Seq was performed as described (Corces et al., 2016) with slight modifications. Briefly, 50,000 cells were collected and centrifuged at 450 x g for 5 minutes. Then, the transposition reaction was performed using digitonin 1%, Tn5 transposase and TD Buffer for 45 minutes at 37°C. Immediately following transposition, the reaction was stopped using a solution of 900 mM NaCl and 300 mM EDTA, 5% SDS and Proteinase K for 30 minutes at 40°C. Transposed DNA fragments were purified using AMPure XP beads, barcoded with Illumina Nextera dual indexes and PCR amplified with KAPA HiFi PCR Kit. Then, the concentration of the library was determined using Qubit 3.0 and size distribution was assessed using Agilent 4200 TapeStation system. Libraries were sequenced on Illumina NextSeq 500 or NovaSeq6000 instruments (single-end, read length 75 bp).

##### ATAC-Seq data processing, peak calling and normalization

Reads were aligned to the mm10 reference genome using BWA aligner (v. 0.7.15) (Li and Durbin, 2009). Bam files were processed using samtools (v.1.4) (Li et al., 2009) and BEDTools (v. 2.24.0) (Quinlan and Hall, 2010) suites. Duplicated reads, reads with mapping quality below 15, unassigned reads or reads mapped on chromosomes Y and M were removed. Peak calling was performed using MACS2 (v. 2.2.1) (Zhang et al., 2008) callpeak function with parameters -g mm–nomodel–shift −100–extsize 200 and setting q-value lower than 1e-5 as cut-off. Obtained peaks were merged using mergeBed function and resulting regions were termed open chromatin regions (OCRs). All regions were annotated using ChIPpeakAnno R package (v 3.16.1) (Zhu et al., 2010) and defined as proximal or distal if located within or beyond 5kb from an annotated TSS, respectively. Read counts were computed using multiBamCov function from BEDTools and normalized with the TMM method using calcNormFactors function. Dispersion was estimated with the estimateDisp function. Differences in signal intensities across different conditions were evaluated fitting a negative binomial generalized linear model on the dataset with glmQLFit function and then performing a quasi-likelihood (QL) F-test with glmQLFTest function. Sample replicates were included in the design as covariates. CPM (counts per million) values were computed for each region with cpm function. For published ATAC-Seq datasets, fastq files were downloaded from GEO repository using fastq-dump from SRA Toolkit (v. 2.8) and processed as described above.

#### Re-analysis of published ChIP-Seq and ATAC-Seq datasets

We downloaded ATAC-Seq datasets form untreated or LPS-treated (6h) BMDMs (GSE67357, WT_unstimulated_ATAC_rep1, WT_unstimulated_ATAC_rep2, WT_LPS_6h_ATAC_rep1, WT_LPS_6h_ATAC_rep2). We downloaded H3K4me3, H3K27ac and PU.1 ChIP-Seq datasets from untreated or LPS-treated (4h) BMDMs (GSE38377, H3K4me3_UT, H3K4me3_LPS_4h, H3K27ac_UT, H3K27ac_LPS_4h, PU.1_UT, PU.1_LPS_4h). We downloaded H3K9me3 from untreated BMDMs (GSE121640, B6N_H3K9me3_ChIP). Samples were processed as described above. Using computeMatrix function from deepTools (v. 2.4.0) suite, we computed mean read coverages (CPM) over intervals of 10bp within larger regions surrounding each TSS of PGE_2_-sensitive, IL-4-sensitive, IL-10-sensitive or resistant genes (TSS ± 1,000bp for ATAC-Seq data and PU.1 ChIP-Seq; TSS ± 3,000bp for H3K4me3 ChIP-Seq; TSS ± 5,000bp for H3K27ac ChIP-Seq).

#### Analysis of LPS-inducible enhancers in BMDMs

##### Definition of LPS-inducible enhancers, sensitive or resistant to costimulation

We obtained a set of 53,925 H2K27ac ChIP-Seq regions after merging peaks from all samples, as described above. We focused on distal H3K27ac regions (n=39,606), which we operationally defined as enhancers. LPS-inducible enhancers were defined by comparing H3K27ac levels (CPM) in the LPS versus UT condition, setting log_2_FC(CPM_LPS_/CPM_UT_) ≥ 2 and FDR < 0.05 as cut-offs. Only regions passing MACS2 q-value cutoff in at least three samples in the comparison were selected, leading to the identification of 2,850 LPS-inducible enhancers. We then classified these regions as PGE_2_-sensitive (n=848) if having log_2_FC(CPM_LPS+PGE2_/CPM_LPS_) ≤ -2 and FDR < 0.05 in the LPS+PGE_2_ versus LPS comparison. Analogously, we defined IL-10-sensitive enhancers (n=1,093) by comparing the LPS+IL-10 to the LPS condition. We then identified PGE_2_-resistant (n=322) and IL-10-resistant (n=306) enhancers setting -0.5 < log_2_FC(CPM_LPS+co-stimulus_/CPM_LPS_) < 0.5 as cut-off.

##### Definition of pre-existing or de novo OCRs within LPS-inducible enhancers.

To define pre-existing OCRs within LPS-inducible enhancers, we first selected OCRs (n=76,203) merging ATAC-Seq peaks passing MACS2 q-value cutoff in all three replicates of UT BMDMs, and then retained those OCRs (n=1,265) mapping within previously defined LPS-inducible enhancers (based on H3K27ac ChIP-Seq). These enhancers were classified as pre-existing enhancers. To define *de novo* OCRs within LPS-inducible enhancers, we first selected OCRs merging ATAC-Seq peaks passing MACS2 q-value cutoff in all three replicates of LPS BMDMs; then, we discarded regions overlapping with previously defined pre-existing OCRs; finally, we retained those OCRs (n=1,476) mapping within previously defined LPS-inducible enhancers. These enhancers were classified as *de novo* enhancers. Read counts were computed on pre-existing or *de novo* OCRs and CPM estimated as described above. We assigned a summit to each *pre-existing* or *de novo* OCR. We retained OCRs in which peak summits from all three replicates (UT or LPS) mapped within 120bp and computed the mean position of summits. These analyses defined two sets of 177 and 221 pre-existing OCRs summits and two sets of 433 and 595 *de novo* OCRs summits within PGE_2_-sensitive and IL-10-sensitive LPS-inducible enhancers respectively; and 176 and 213 pre-existing OCRs summits and 182 and 119 *de novo* OCRs summits within PGE_2_-resistant and IL-10-resistant LPS-inducible enhancers respectively.

##### PU.1 occupancy within LPS-inducible enhancers, sensitive or resistant to costimulation

We computed PU.1 read counts on pre-existing and *de novo* OCRs summits (+/- 100bp) within PGE_2_-sensitive and IL-10-sensitive or PGE_2_-resistant IL-10-resistant enhancers; as well as number of overlapping peaks for the untreated BMDMs.

##### TF binding in pre-existing or de novo OCRs within LPS-inducible enhancers.

We used computeMatrix function from deepTools (v. 2.4.0) (Ramirez et al., 2014) suite to compute mean read coverages (CPM) within previously defined OCR summits +/- 1,000bp. Heatmap was produced with plotHeatmap function. For all transcription factors, we also reported cpm computed over pre-existing and *de novo* OCRs summits (+/- 100bp) within PGE_2_-sensitive or PGE_2_-resistant enhancers.

##### Chromatin state discovery and characterization of BMDMs

We downloaded H3K4me1, H3K4me3, H3K9me3, H3K9ac, H3K36me3 and CTCF ChIP-seq data from untreated BMDMs (GSE38377, H3K4me1_UT and H3K4me3_UT. GSE121640, B6N_H3K9me3_ChIP. GSE113226, WT_BMDM_UT_H3K9ac_ChIP-seq. GSE125159, H3K36me3-0. GSE107455, Genome-wide localization analyses of CTCF (Untreated) binding sites). Samples were aligned and processed as described above and then bam files were converted into binarized data files using BinarizeBam function from ChromHMM tool (v 1.22). Finally, LearnModel function was applied setting the number of states to 7.

##### Motif enrichment analysis within OCRs

We first defined genomic regions spanning 200bp from previously defined OCRs summits and performed analyses with HOMER (v. 4.10) (Heinz et al., 2010), using the findMotifsGenome.pl script with parameters -size given -mask -h.

#### Analysis of LPS-inducible enhancers in wt or MEF2A-deficient iMacs

##### Definition of LPS-inducible enhancers in iMacs

Following the procedure described above for H3K27ac ChIP-Seq on BMDMs, we defined a set of 42,251 regions (29,596 distal) merging peaks from all samples. To estimate differences in signal intensities in these regions, MEF2A-deficient iMac clones (D7, A7, A8, C7) and MEF2A-proficient (referred to as wt) iMac clones (NE, B3 and D10) were considered as sets of biological replicates. LPS-inducible enhancers were defined in wt iMacs by comparing H3K27ac levels (CPM) in the LPS versus UT condition, setting log_2_FC(CPM_LPS_/CPM_UT_) ≥ 2 and FDR < 0.05 as cut-offs. Only distal regions passing MACS2 q-value cutoffs in at least two samples in the comparison were selected, leading to the definition of 3,421 LPS-inducible enhancers in iMacs. We then classified these regions as MEF2A-dependent (n=981) if having log_2_FC(CPM_MEF2Ako_LPS_/CPM_WT_LPS_) ≤ -2 and FDR < 0.05 in MEF2A-deficient vs wt LPS treated iMacs comparison. As a control group of MEF2A-independent enhancers, we selected 916 regions whose acetylation in response to LPS was not affected in MEF2A-deficient as compared to wt iMacs, setting -0,5 < log_2_FC(CPM_MEF2Ako_LPS_/CPM_WT_LPS_) < 0,5 as cut-offs.

Analogously, we defined MEF2A-dependent enhancers in untreated iMacs (n=998), setting log_2_FC(CPM_MEF2Ako_UT_/CPM_WT_UT_) ≤ -2 and FDR < 0.05 and selecting distal regions passing MACS2 q-value cutoffs in at least two untreated samples (Basal enhancers). As a control group of MEF2A-independent enhancers, we selected 8545 regions passing MACS2 q-value cutoffs in at least two untreated samples and whose acetylation was not affected in MEF2A-deficient as compared to wt untreated iMacs, setting -0,5 < log_2_FC(CPM_MEF2Ako_UT_/CPM_WT_UT_) < 0,5 as cut-offs.

We then focused on PGE_2_-sensitive (n=551) or PGE_2_-resistant enhancers (n=190) that were LPS-inducible in both BMDMs and iMacs. Read counts were computed and CPM values estimated as described above. We also computed CPM values for BMDMs on MEF2A-dependent (LPS) (n=669) and MEF2A-independent (LPS) (n=377) enhancers that were LPS-inducible in both BMDMs and iMacs.

##### Definition of pre-existing or de novo OCRs in iMacs

ATAC-Seq data were processed as described above. We defined pre-existing OCRs merging ATAC-Seq peaks passing MACS2 q-value cutoff in all replicates of UT wt iMacs. To define *de novo* OCRs we first merged ATAC-Seq peaks passing MACS2 q-value cutoff in all four replicates of LPS wt iMacs; then, we discarded regions overlapping with previously defined pre-existing OCRs. We then assigned a summit to each *pre-existing* or *de novo* OCR as described above for BMDMs. These analyses defined two sets of 279 and 3500 pre-existing OCRs summits within MEF2A-dependent (UT) and MEF2A-independent (UT) basal enhancers, respectively; and two sets of 404 and 628 *de novo* OCRs summits within MEF2A-dependent (LPS) and MEF2A-independent (LPS) LPS-inducible enhancers, respectively.

CPM were computed on pre-existing and *de novo* OCRs (previously defined in BMDMs) within PGE_2_-sensitive or PGE_2_-resistant enhancers that were LPS-inducible in both BMDMs and iMacs.

##### Motif enrichment analysis within OCRs

We first defined genomic regions spanning 200bp from previously defined OCRs summits and performed analyses with HOMER (v. 4.10) (Heinz et al., 2010), using the findMotifsGenome.pl script with parameters -size given -mask -h. We compared motif enrichment within MEF2A-dependent versus MEF2A-independent enhancers. For MEF2A-independent basal enhancers we selected the top 1000 regions with lower absolute values of logFC(CPM_MEF2Ako_UT_/CPM_WT_UT_) in order to have a comparable number of regions with basal MEF2A-dependent enhancers.

##### PU.1 signal intensity on pre-existing or de novo OCRs within LPS-inducible enhancers in iMacs

We computed PU.1 read counts from all samples on *pre-existing* and *de novo* OCRs summits (+/- 100bp) (defined in BMDMs) within PGE_2_-sensitive or PGE_2_-resistant enhancers that were LPS-inducible in both BMDMs and iMacs.

### Quantification and statistical analysis

Results are illustrated as mean ± SD. Graphs show data from at least two independent repeats. Significance was defined as p < 0.05. Statistical analysis was conducted either using GraphPad Prism v9.0 (GraphPad Software) or R v3.4.1 (R project). The specific statistical tests, exact value of n, what n represents are mentioned in the figure legends.

## Supplemental information

Document S1. Figures S1–S7

Table S1. Bulk RNA-seq analysis in BMDMs, related to Figures 1, S1, and S4 List of LPS-induced or IFN-α-induced genes in BMDMs and their classification as sensitive or resistant to costimulation with PGE_2_, IL-10 or IL-4. PGE_2_-induced, IL-10-induced or IL-4-induced genes are also reported. List and expression values of PGE_2_-sensitive and resistant genes in BMDMs treated or not with IFN-β or anti-IL-10R antibody; classification of genes as restored, partially restored or not restored is reported. Effect of BRD2-4 or CBP-p300 inhibition on LPS-induced genes that are sensitive or resistant to costimulation with PGE_2_, IL-10 or IL-4. Data used for heatmaps shown in Figure 1A and Figure S1I.

Table S2. scRNA-seq data analysis of BMDMs, related to Figures 2 and S2 Size and composition of scRNA-Seq clusters. scRNA-Seq data expression values of cluster-specific genes and LPS-induced, PGE_2_-induced or IFN-α-induced genes defined from bulk RNA-Seq data.

Table S3. ChIP-seq and ATAC-seq analysis in BMDMs, related to Figures 4, S4, and S7 List and CPM values of LPS-inducible enhancers and their classification as sensitive or resistant to costimulation with PGE_2_ or IL-10. PU.1 ChIP-Seq or ATAC-Seq intensities are reported on overlapping pre-existing and *de novo* OCRs.

Table S4. TF ChIP-seq and motif enrichment analysis in BMDMs, related to Figure 5, S5, and S7 List and features of TF ChIP-Seq peaks at pre-existing or *de novo* OCRs within PGE_2_-sensitive or resistant enhancers. Motif enrichment analyses related to Figures 5A, S7R, and S7S.

Table S5. ChIP-seq, ATAC-seq, and motif enrichment analysis in iMacs, related to Figures 5 and S5 List and signal intensities for H3K27ac for MEF2A-dependent or MEF2A-independent basal and LPS-inducible enhancers in wt or *Mef2a*^*−/−*^ iMacs and in BMDMs, untreated or stimulated with LPS. Signal intensities for H3K27ac or PU.1 ChIP-Seq as well as ATAC-Seq for PGE_2_-sensitive or resistant enhancers in wt or *Mef2a*^*−/−*^ iMacs, untreated or stimulated with LPS. Motif enrichment analyses related to Figure 5E.

Table S6. RNA-seq in MEF2 TF-deficient iMacs and BMDMs, related to Figure 6 List and expression values of LPS-induced genes in wt or *Mef2a*^*−/−*^ iMacs and wt or MEF2C-D double-deficient BMDMs. Classification of genes as MEF2A-dependent or MEF2A-independent is reported.

Table S7, Sanger sequencing and list of oligonucleotides, related to Figures 6, 7, and S6 and STAR Methods Sanger sequencing data of iMacs clones generated in this study. List and sequences of oligonucleotides used in this study.

Document S2. Article plus supplemental information
