# Supplementary material for: A PGE2-MEF2A axis enables context-dependent control of inflammatory gene expression
Source: Immunity. 2021 Aug 10;54(8):1665–1682.e14. doi: 10.1016/j.immuni.2021.05.016 (PMC8362890; doi:10.1016/j.immuni.2021.05.016)
Supplement: Document S1. Figures S1–S7 [file mmc1.pdf]

Immunity, Volume *54*

Supplemental information

A PGE_2-MEF2A axis enables context-dependent control of inflammatory gene expression

Francesco Cilenti, Giulia Barbiera, Nicoletta Caronni, Dario Iodice, Elisa Montaldo, Simona Barresi, Eleonora Lusito, Vincenzo Cuzzola, Francesco Maria Vittoria, Luca Mezzanzanica, Paolo Miotto, Pietro Di Lucia, Dejan Lazarevic, Daniela Maria Cirillo, Matteo Iannacone, Marco Genua, and Renato Ostuni

SUPPLEMENTAL INFORMATION

A PGE_2-MEF2A axis enables context-dependent control of inflammatory gene expression

Francesco Cilenti[1,2,3,8], Giulia Barbiera[2,3,8], Nicoletta Caronni[2,3], Dario Iodice[2,3], Elisa Montaldo[2,3], Simona Barresi[2,3], Eleonora Lusito[2,3], Vincenzo Cuzzola[2,3], Francesco Maria Vittoria[1,2,3], Luca Mezzanzanica[1,2,3], Paolo Miotto[4], Pietro Di Lucia[5], Dejan Lazarevic[6], Daniela Maria Cirillo[4], Matteo Iannacone[1,5,7], Marco Genua[2,3,9] and Renato Ostuni[1,2,3,9,10]

[1]Vita-Salute San Raffaele University, Milan, Italy

[2]San Raffaele Telethon Institute for Gene Therapy (SR-Tiget)

[3]Genomics of the Innate Immune System Unit, IRCCS San Raffaele Scientific Institute, Milan, Italy

[4]Emerging Bacterial Pathogens Unit, Division of Immunology, Transplantation and Infectious Diseases, IRCCS San Raffaele Scientific Institute, Milan, Italy

[5]Dynamics of Immune Responses Unit, Division of Immunology, Transplantation and Infectious Diseases, IRCCS San Raffaele Scientific Institute, Milan, Italy

[6]Center for Omics Sciences (COSR), IRCCS San Raffaele Scientific Institute, Milan, Italy

[7]Experimental Imaging Centre, IRCCS San Raffaele Scientific Institute, Milan, Italy

[8]These authors contributed equally

[9]These authors contributed equally

[10]Corresponding author and Lead Contact (ostuni.renato@hsr.it)

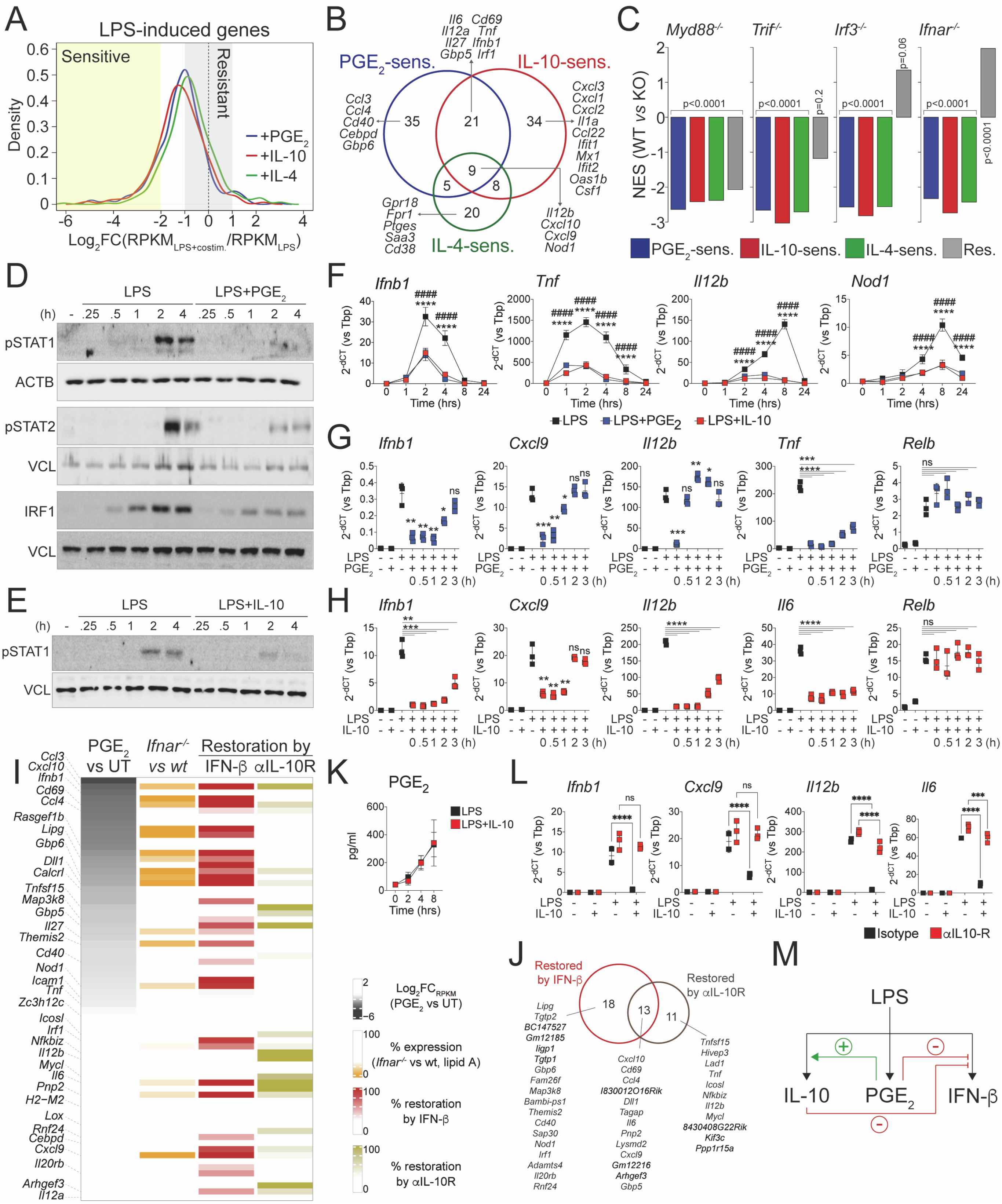

Figure S1. Impact of costimulation on LPS-induced gene expression, Related to Figure 1. (A) Density plot showing the effect of PGE_2, IL-10, or IL-4 on LPS-induced gene expression. Dotted line indicates lack of effect of the costimulation; yellow or grey shaded areas indicate values used to define costimulation-sensitive or resistant genes, respectively. Data from two biological replicates. Pearson

correlation > 0.97 for all replicates. (B) Overlap between PGE_2-sensitive (blue), IL-10-sensitive (red) or IL-4-sensitive (green) genes. Selected gene names are shown. (C) Gene Set Enrichment Analysis (GSEA) of PGE_2-sensitive (blue), IL-10-sensitive (red), IL-4-sensitive (green) or resistant (grey) transcripts (gene sets) in ranked gene lists obtained comparing lipid A-stimulated *Myd88*$^{-/-}$, *Ticam1*$^{-/-}$, *Irf3*$^{-/-}$, *Ifnar1*$^{-/-}$ versus wt BMDMs (data from Tong et al., Cell 2016). Normalized enrichment score (NES) and p-values are shown for each plot. (D) Western blot analyses for phosphorylated STAT1 (Tyr701), STAT2 (Tyr689), IRF1 and loading controls in BMDMs stimulated with LPS or LPS+PGE_2 for the indicated time points. (E) Western blot analyses for phosphorylated STAT1 (Tyr701) in BMDMs stimulated with LPS or LPS+IL-10 for the indicated time points. (F) RT-qPCR analysis of a set of PGE_2- and IL-10-sensitive genes in BMDMs stimulated as indicated. Line plot represents mean ± SD. Data from three biological replicates. **** p<0.0001 LPS vs LPS+PGE_2, #### p<0.0001 LPS vs LPS+IL-10 (two-way ANOVA test). (G-H) RT-qPCR analysis of a set of sensitive genes in BMDMs stimulated with LPS in the absence or presence of PGE_2 (G) or IL-10 (H), given after the inflammatory stimulus at the indicated time point. Line plots represent mean ± SD. Data from three biological replicates. **** p<0.0001, *** p<0.001, ** p<0.01, * p<0.05, ns not significant (unpaired *t*-test). (I) Heatmap showing the behavior of PGE_2-sensitive genes. Genes are ranked by ascending values of log_2FC_{RPKM} in the PGE_2 vs UT condition (left lane, grey scale). Right lanes represent the percentage of gene expression in lipid A-stimulated *Ifnar1*$^{-/-}$ vs wt BMDMs (data from Tong et al., Cell 2016) (orange scale), as well as percentage of restoration by IFN-β (red scale) or anti-IL-10R antibody (brown scale) treatment in BMDMs costimulated with LPS+PGE_2. Selected gene names are shown on the left, legends are shown on the right. Data from two or three biological replicates. Pearson correlation > 0.97 for all replicates. (J) Overlap between PGE_2-sensitive genes restored by exogenous IFN-β or by anti-IL-10R antibody treatment in costimulated BMDMs. (K) PGE_2 release by BMDMs stimulated as indicated. Line plot represents mean ± SD. Data from three biological replicates. (L) RT-qPCR analysis of a set of IL-10 sensitive genes in BMDMs stimulated as indicated. Dot plots represent mean ± SD. Data from three biological replicates. **** p<0.0001, *** p<0.001, ns, not significant; (two-way ANOVA test). (M) Regulatory circuits elicited by PGE_2 in costimulated macrophages.

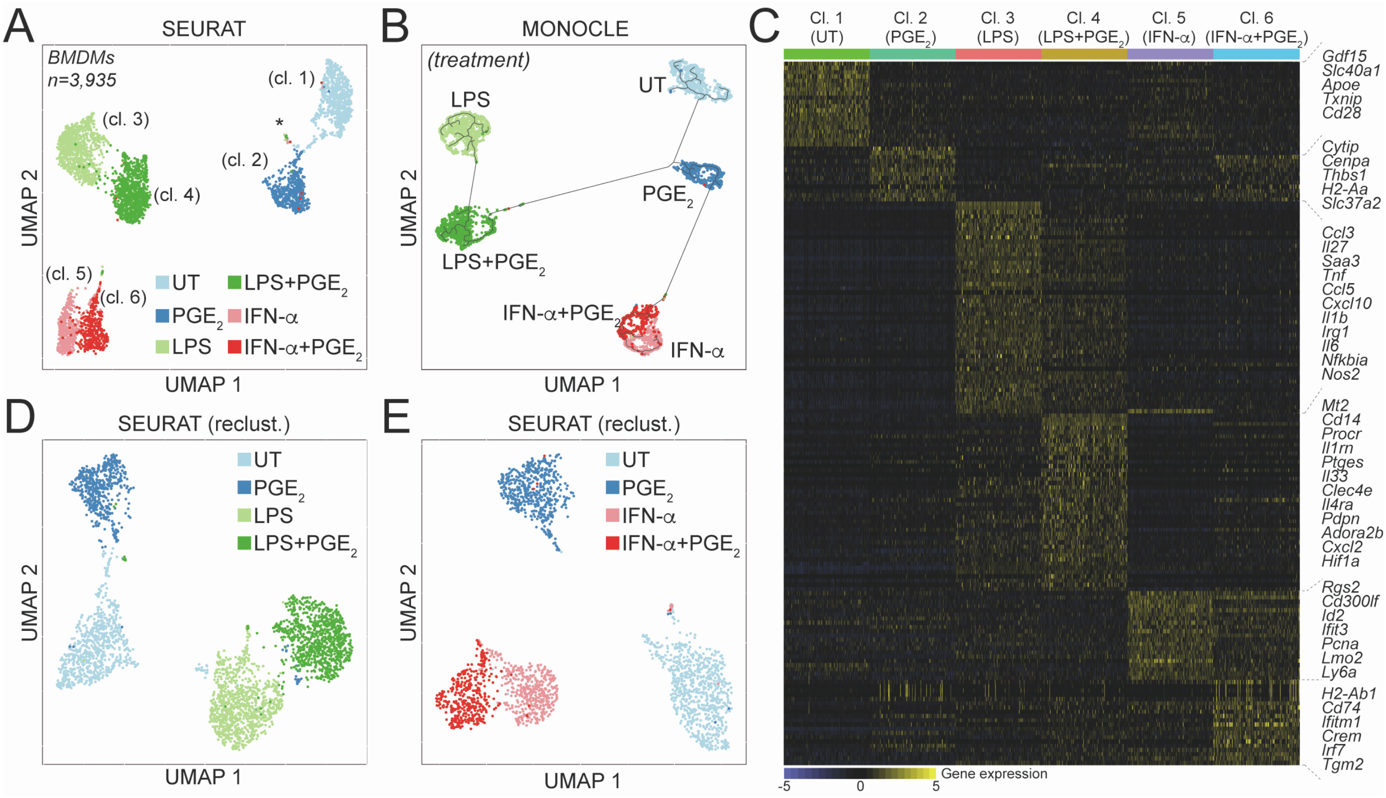

Figure S2. Single-cell RNA-Seq analysis in costimulated BMDMs, Related to Figure 2. (A) UMAP plot of scRNA-Seq BMDMs stimulated as indicated. Colors represent experimental conditions; corresponding clusters are shown in brackets. The asterisk indicates a small cluster (n=35) of contaminant cells that were excluded from analyses. (B) Monocle3 analysis (see STAR Methods) of scRNA-Seq data from stimulated BMDMs, color-coded by type of treatment. (C) Expression values (log-normalized UMI counts) of a set of genes for each cluster. (D-E) UMAP plot showing re-clustering of BMDMs from UT, PGE_2, LPS, LPS+PGE_2 (D) or UT, PGE_2, IFN-α, IFN-α+PGE_2 (E) conditions, color-coded by type of treatment.

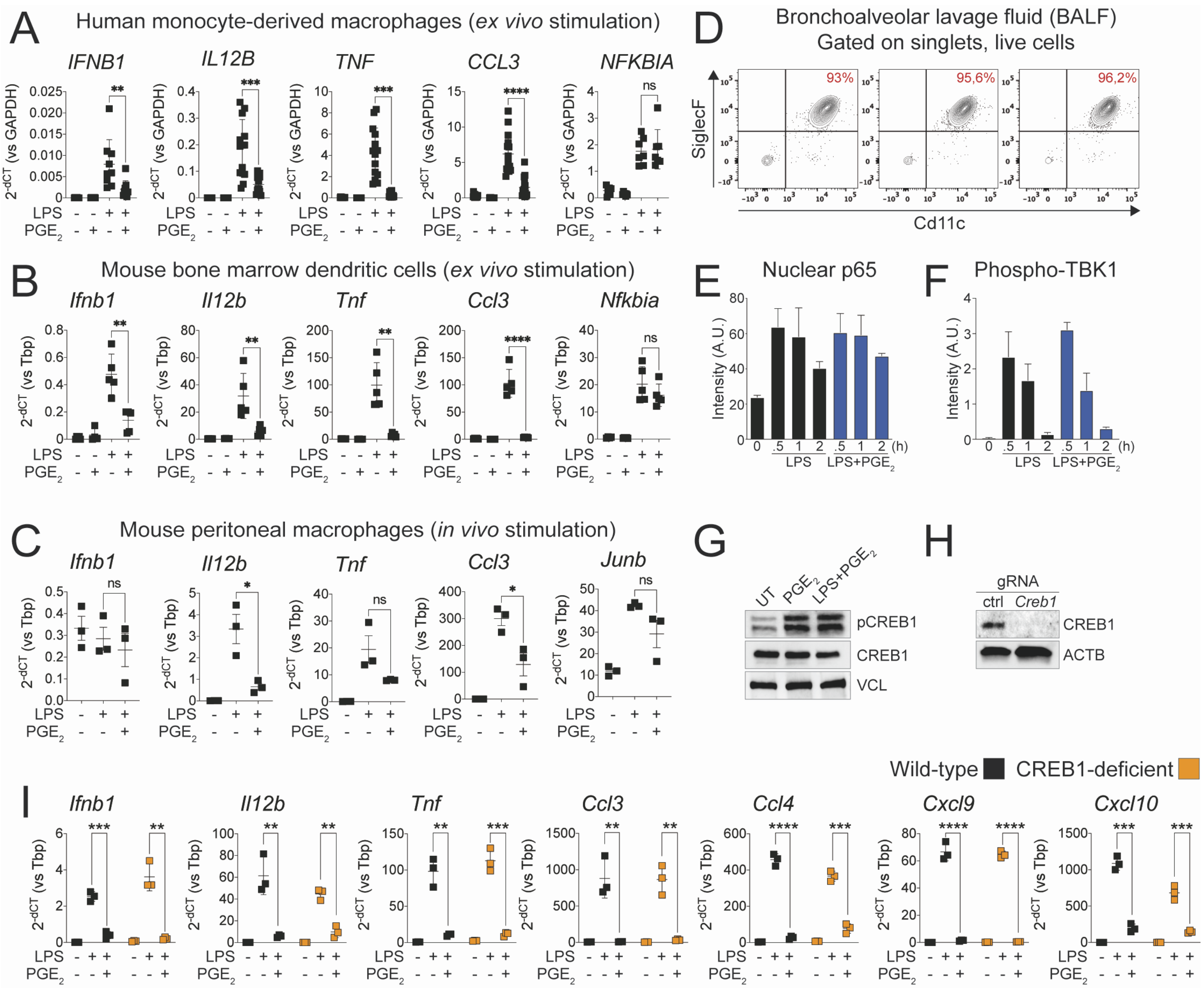

Figure S3. Impact of PGE_2 on LPS-induced gene expression and signaling. Related to Figure 3. (A-C) RT-qPCR analysis of a set of PGE_2-sensitive or resistant genes in human monocyte-derived macrophages (A), mouse bone marrow-derived dendritic cells (B), or mouse peritoneal macrophages (C) stimulated as indicated. Dot plots represent mean ± SD. Data from seven (A), five (B) or three (C) biological replicates. **** p<0.0001, *** p<0.001, ** p<0.01, * p<0.05, ns not significant (unpaired *t*-test). (D) Selected flow cytometry plots showing the expression of Cd11c and SiglecF in BALF cells of untreated mice. (E-F) Immunofluorescence analyses of nuclear NF-κB p65 (E) or phosphorylated TBK1 (F) in BMDMs stimulated as indicated. Bar plots represent mean ± SD. Data from three biological replicates. (G) Western blot analyses for phosphorylated CREB1 (Ser133) in BMDMs stimulated with PGE_2 or LPS+PGE_2 for 30 minutes. CREB1 and Vinculin (VCL) are reported as loading controls. (H) Western blot analysis for CREB1 in BMDMs upon CRISPR-Cas9-mediated targeting of *Creb1*. β-Actin (ACTB) is reported as loading control. (I) RT-qPCR analysis of a set of PGE_2-sensitive genes in wt (black) and CREB1-deficient (orange) BMDMs. Dot plots represent mean ± SD. Data from three biological replicates. **** p<0.0001, *** p<0.001, ** p<0.01, (unpaired *t*-test).

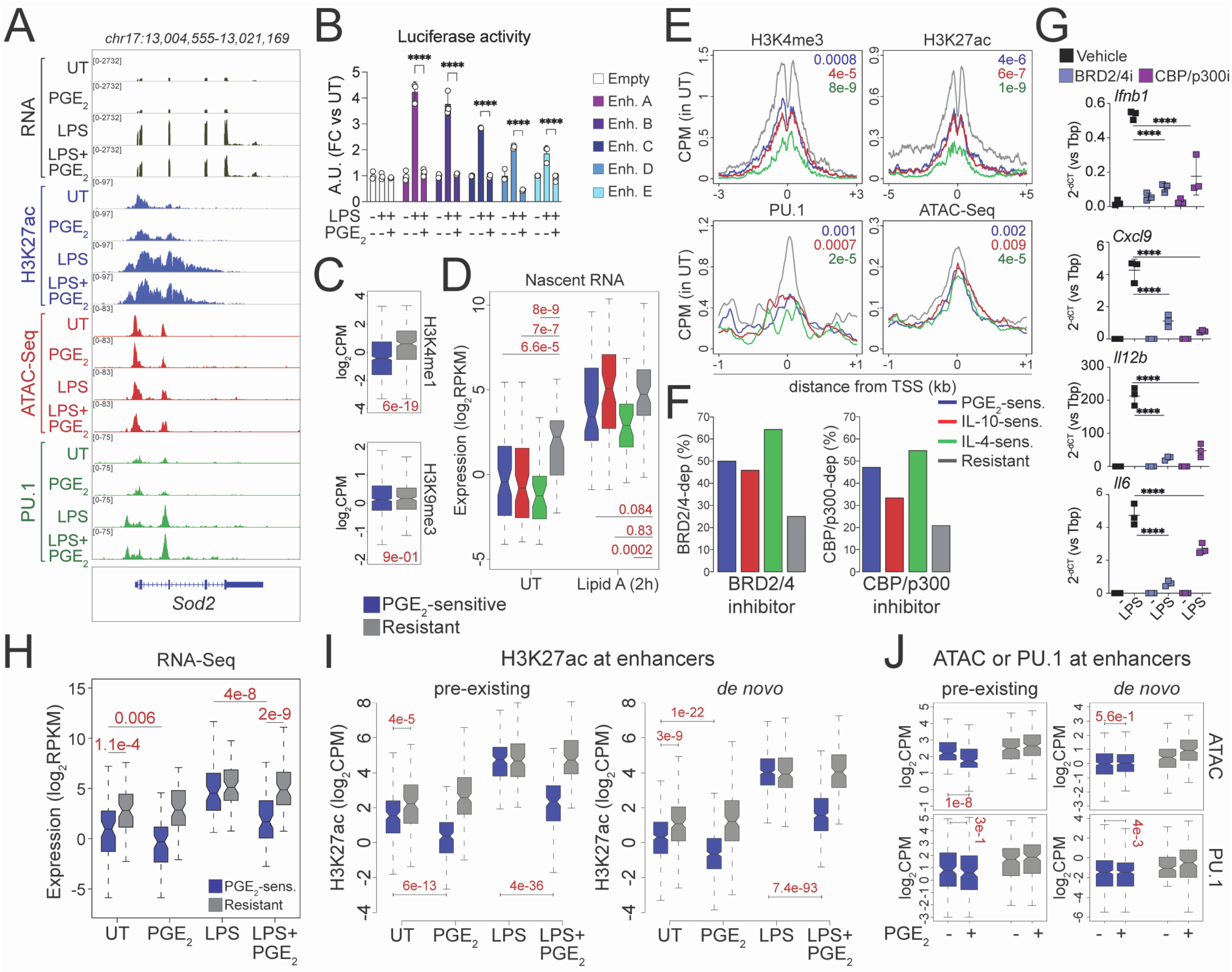

Figure S4. Genomic properties of PGE_2-sensitive enhancers, Related to Figure 4. (A) IGV snapshot showing read coverage of the indicated datasets at a selected PGE_2-resistant genomic locus in co-stimulated BMDMs. (B) Functional validation of selected PGE_2-sensitive enhancers in a luciferase reporter assay. A.U. = arbitrary unit. Bar plots represent mean ± SD. Data from three biological replicates. **** $p<0.0001$, (two-way ANOVA test). (C) Box plot showing H3K4me1 (top) or H3K9me3 (bottom) ChIP-Seq signal intensity in unstimulated BMDMs of PGE_2-sensitive (blue) or resistant (grey) enhancers (data from Ostuni et al., Cell 2013 and Treger et al., Immunity 2019). Numbers indicate p-values (Mann-Whitney U test) for the indicated comparisons. (D) Mean expression values of chromatin-associated RNA (Tong et al., Cell 2016) of PGE_2-sensitive (blue), IL-10-sensitive (red), IL-4-sensitive (green) or resistant (grey) genes in untreated and lipid A-stimulated BMDMs. Numbers indicate p-values (Mann-Whitney U test) for the indicated comparisons. (E) Mean intensities (CPM) of H3K4me3, H3K27ac, PU.1 ChIP-Seq or ATAC-Seq signals in genomic regions spanning the transcription start site (TSS) of PGE_2-sensitive (blue), IL-10-sensitive (red), IL-4-sensitive (green) or resistant (grey) genes in unstimulated BMDMs (data from Ostuni et al., Cell 2013 and Cuartero et al., Nat Immunol 2018). Numbers indicate p-values (Mann-Whitney U

test) comparing coverages (CPM) computed on the displayed genomic regions. (F) Percentage of PGE_2-sensitive (blue), IL-10-sensitive (red), IL-4-sensitive (green) or resistant (grey) genes whose induction by LPS was reduced (see STAR Methods) by inhibition of BRD2-4 (left) or CBP-p300 (right). Data from three biological replicates. Pearson correlation > 0.98 for all replicates. (G) RT-qPCR analysis of a set of PGE_2-sensitive genes in BMDMs stimulated with LPS in the absence or presence of BRD2-4 or CBP-p300 inhibitors. Dot plots represent mean $\pm$ SD. Data from three biological replicates. (H) Mean expression values of PGE_2-sensitive (blue) or resistant (grey) genes in the indicated conditions. Data from two biological replicates. Pearson correlation > 0.97 for all replicates. Numbers indicate p-values for the corresponding comparisons (Mann-Whitney U test). (I) H3K27ac ChIP-Seq mean signal intensity in pre-existing (left) or *de novo* (right) OCRs at PGE_2-sensitive or resistant enhancers in the indicated conditions. Data from two biological replicates. Pearson correlation > 0.94 for all replicates. Numbers indicate p-values (Mann-Whitney U test) for the corresponding comparisons. (J) ATAC-Seq (top) and PU.1 ChIP-Seq (bottom) signal intensities within pre-existing (left) or *de novo* (right) OCRs (see Methods) at PGE_2-sensitive or resistant enhancers in the indicated conditions. Numbers indicates p-values (Mann-Whitney U test) for the corresponding comparisons.

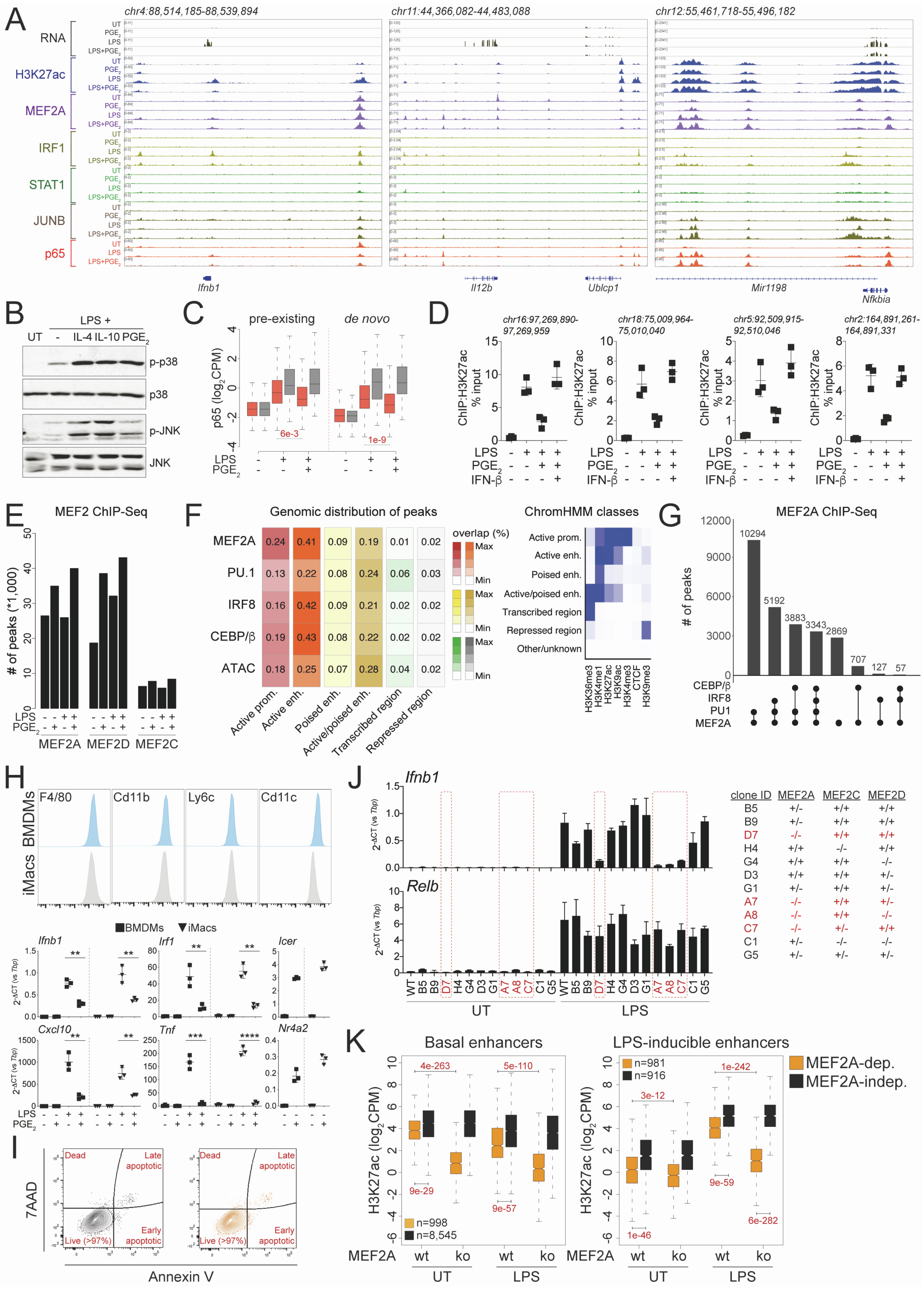
A
RNA
H3K27ac
MEF2A
IRF1
STAT1
JUNB
p65
chr4:88,514,185-88,539,894
chr11:44,366,082-44,483,088
chr12:55,461,718-55,496,182
Ifnb1
Il12b
Ublcp1
Mir1198
Nfkbia
B
LPS +
UT - IL-4 IL-10 PGE2
p-p38
p38
p-JNK
JNK
C
pre-existing
de novo
p65 (log2CPM)
6e-3
1e-9
D
ChIP:H3K27ac % input
E
MEF2 ChIP-Seq
# of peaks (*1,000)
MEF2A MEF2D MEF2C
F
Genomic distribution of peaks
MEF2A 0.24 0.41 0.09 0.19 0.01 0.02
PU.1 0.13 0.22 0.08 0.24 0.06 0.03
IRF8 0.16 0.42 0.09 0.21 0.02 0.02
CEBP/β 0.19 0.43 0.08 0.22 0.02 0.02
ATAC 0.18 0.25 0.07 0.28 0.04 0.02
Active prom. Active enh. Poised enh. Active/poised enh. Transcribed region Repressed region
overlap (%)
ChromHMM classes
Other/unknown
G
MEF2A ChIP-Seq
# of peaks
10294 5192 3883 3343 2869 707 127 57
CEBP/β
IRF8
PU1
MEF2A
H
F4/80 Cd11b Ly6c Cd11c
BMDMs
iMacs
Ifnb1 Irf1 Icer Cxcl10 Tnf Nr4a2
J
Ifnb1
Relb
clone ID MEF2A MEF2C MEF2D
B5 +/- +/+ +/+
B9 +/- +/+ +/+
D7 -/- +/+ +/+
H4 +/+ -/- +/+
G4 +/+ +/+ -/-
D3 +/+ +/+ +/-
G1 +/- +/+ +/-
A7 -/- +/+ +/-
A8 -/- +/+ -/-
C7 -/- +/- +/+
C1 +/- -/- -/-
G5 +/- +/- +/-
UT
LPS
I
7AAD
Annexin V
Dead
Late apoptotic
Live (>97%)
Early apoptotic
K
Basal enhancers
LPS-inducible enhancers
H3K27ac (log2CPM)
4e-263 5e-110 9e-29 9e-57
n=998
n=8,545
n=981
n=916
3e-12 1e-242 1e-46 9e-59 6e-282
MEF2A-dep.
MEF2A-indep.
MEF2A wt ko wt ko

Figure S5. Role of MEF2 TFs in the control of macrophage enhancers, Related to Figure 5. (A) IGV snapshots showing read coverage of the indicated datasets at selected PGE_2-sensitive (left panels) or resistant (right panel) genomic loci in costimulated BMDMs. (B) Western blot analyses in BMDMs for phosphorylated p38 (Thr180/Tyr182) or JNK (Thr183/Tyr185) as well as non-phosphorylated forms in the indicated conditions. Data shown refer to a stimulation of 30 minutes. (C) p65 ChIP-Seq signal intensities at pre-existing or *de novo* OCRs within PGE_2-sensitive (orange) or resistant (grey) enhancers in the indicated conditions. Numbers denote p-values (Mann-Whitney U test) for the indicated comparisons. (D) ChIP-qPCR analysis of a set of PGE_2-sensitive enhancers in BMDMs stimulated as indicated. Dot plots represent mean ± SD. Data from three biological replicates. (E) Number of ChIP-Seq peaks identified for MEF2A, MEF2D or MEF2C in BMDMs stimulated as indicated. (F) Percentage of TF ChIP-Seq peaks that overlap with the indicated chromatin classes estimated by ChromHMM (right, see STAR Methods). (G) Number of MEF2A ChIP-Seq peaks overlapping with the indicated TFs. (H) Surface expression (top panels) of the indicated markers in differentiated BMDMs (light blue) or iMacs (grey). RT-qPCR analysis (bottom panels) of selected LPS-induced or PGE_2-induced genes in wt BMDMs or iMacs in the indicated conditions. Dot plots represent mean ± SD. Data from three biological replicates. **** $p< 0.0001$, *** $p< 0.001$, ** $p< 0.01$ (unpaired *t*-test). (I) Annexin V and 7AAD staining in wt (black) or MEF2A-deficient (orange) iMacs. Data from one of three biological replicates. (J) RT-qPCR analysis (left) of *Ifnb1* and *Relb* on clones of iMacs in the indicated conditions. MEF2A-deficient clones are highlighted in red. The ID and genotype of each clone are shown on the right. (K) H3K27Ac mean intensity values for basal (left) or LPS-inducible (right) MEF2A-dependent (orange) and MEF2A-independent (black) enhancers (see STAR Methods). Data are shown for wt or MEF2A-deficient iMacs in the indicated conditions. Data from three biological replicates. Pearson correlation > 0.94 for all replicates. Numbers indicate p-values (Mann-Whitney U test) for the corresponding comparisons.

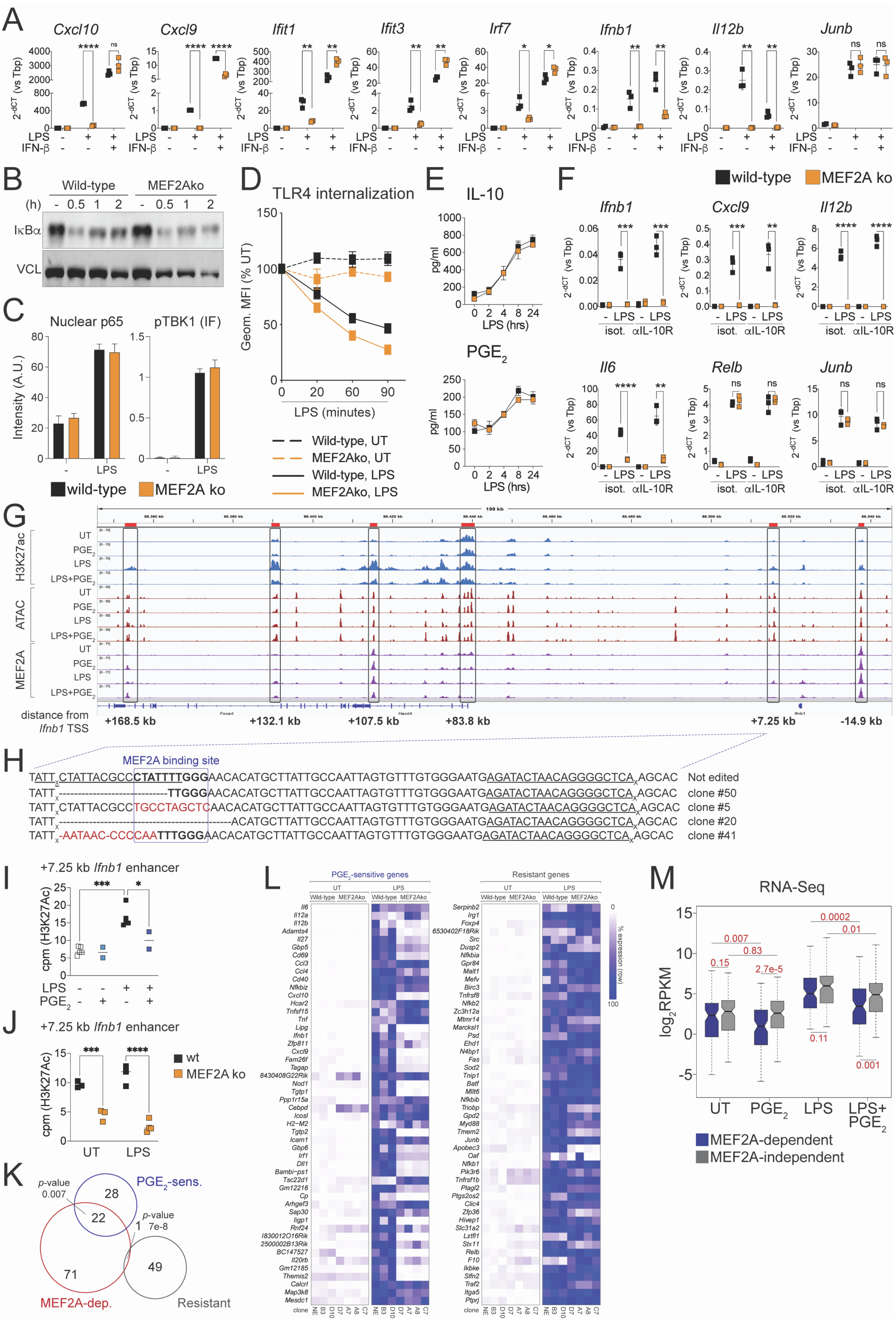
A
Cxcl10
Cxcl9
Ifit1
Ifit3
Irf7
Ifnb1
Il12b
Junb
LPS
IFN-β
B
Wild-type
MEF2Ako
IκBα
VCL
C
Nuclear p65
pTBK1 (IF)
Intensity (A.U.)
wild-type
MEF2A ko
D
TLR4 internalization
Geom. MFI (% UT)
LPS (minutes)
Wild-type, UT
MEF2Ako, UT
Wild-type, LPS
MEF2Ako, LPS
E
IL-10
PGE2
pg/ml
LPS (hrs)
F
wild-type
MEF2A ko
Ifnb1
Cxcl9
Il12b
Il6
Relb
Junb
isot.
αIL-10R
G
H3K27ac
ATAC
MEF2A
UT
PGE2
LPS
LPS+PGE2
distance from Ifnb1 TSS
+168.5 kb
+132.1 kb
+107.5 kb
+83.8 kb
+7.25 kb
-14.9 kb
H
MEF2A binding site
TATTxCTATTACGCCCTATTTTGGGAACACATGCTTATTGCCAATTAGTGTTTGTGGGAATGAGATACTAACAGGGGCTCAxAGCAC Not edited
TATTx--------------------------TTGGGAACACATGCTTATTGCCAATTAGTGTTTGTGGGAATGAGATACTAACAGGGGCTCAxAGCAC clone #50
TATTxCTATTACGCCTGCCTAGCTCAACACATGCTTATTGCCAATTAGTGTTTGTGGGAATGAGATACTAACAGGGGCTCAxAGCAC clone #5
TATTx------------------------------------ACATGCTTATTGCCAATTAGTGTTTGTGGGAATGAGATACTAACAGGGGCTCAxAGCAC clone #20
TATTx-AATAAC-CCCCAATTTGGGAACACATGCTTATTGCCAATTAGTGTTTGTGGGAATGAGATACTAACAGGGGCTCAxAGCAC clone #41
I
+7.25 kb Ifnb1 enhancer
cpm (H3K27Ac)
J
+7.25 kb Ifnb1 enhancer
wt
MEF2A ko
UT
LPS
K
p-value 0.007
PGE2-sens.
28
22
p-value 7e-8
1
71
49
MEF2A-dep.
Resistant
L
PGE2-sensitive genes
Resistant genes
UT
LPS
Wild-type
MEF2Ako
% expression (row)
clone
M
RNA-Seq
log2RPKM
UT
PGE2
LPS
LPS+PGE2
MEF2A-dependent
MEF2A-independent

Figure S6. Role of MEF2A in the control of LPS-induced gene expression and signaling, Related to Figure 6. A) RT-qPCR analysis of a set of PGE_2-sensitive or resistant genes in wt (black) or *Mef2a*$^{-/-}$ (orange) iMacs stimulated as indicated. Dot plots represent mean ± SD. Data from three biological replicates. **** $p< 0.0001$, ** $p< 0.01$, * $p<0.05$, ns not significant (unpaired *t*-test). (B) Western blot analyses of IκBα and loading control (VCL) in whole cell extracts of wt or *Mef2a*$^{-/-}$ iMacs stimulated with LPS for the indicated time points. (C) Immunofluorescence analyses of nuclear NF-κB p65 (left) or phosphorylated TBK1 (right) in wt (black) or *Mef2a*$^{-/-}$ (orange) iMacs stimulated with LPS for 30 minutes. Bar plots represent mean ± SD. Data from three biological replicates. (D) Flow cytometry analysis of TLR4 internalization showing percentage of geometric Mean Fluorescence Intensity relative to the untreated condition in wt (black) or *Mef2a*$^{-/-}$ (orange) iMacs stimulated with control or LPS for the indicated time points. Line plot represents mean ± SD. Data from three biological replicates. (E) IL-10 (top) or PGE_2 (bottom) release by wt (black) or *Mef2a*$^{-/-}$ (orange) iMacs stimulated for the indicated time points with LPS. Line plots represent mean ± SD. Data from three biological replicates. (F) RT-qPCR analysis of a set of PGE_2-sensitive or resistant genes in wt (black) or *Mef2a*$^{-/-}$ (orange) iMacs, stimulated as indicated in the presence or absence of IL10R blocking antibody. Dot plots represent mean ± SD. Data from three biological replicates. **** $p< 0.0001$, *** $p< 0.001$, ** $p<0.01$, ns not significant (unpaired *t*-test). (G) IGV snapshot showing read coverage of the indicated datasets at the *Ifnb1* locus. Enhancers selected for editing are shown. (H) Sequences of control and edited iMac clones encompassing the MEF2A binding site (bold) within the +7.25 kb *Ifnb1* enhancer. Clone IDs are reported. (I-J) H3K27ac mean intensity values in BMDMs (I) or wt (black) and *Mef2a*$^{-/-}$ (orange) iMacs (J) stimulated as indicated. **** $p<0.0001$, *** $p< 0.001$, * $p<0.05$ (two-way ANOVA test). (K) Venn diagram showing the overlap between MEF2A-dependent (red), PGE_2-sensitive (blue) or resistant (grey) genes (see STAR Methods). p-values (hypergeometric test) for the indicated overlaps are shown. (L) Heatmap showing the behavior of PGE_2-sensitive or resistant genes (see STAR Methods) in wt and MEF2A-deficient iMacs in the indicated conditions. Colors represent row-normalized percentage of gene expression across the UT or LPS condition. Gene names, color legend and clone IDs are reported. (M) Mean expression values of MEF2A-dependent (blue) or MEF2A-independent (grey) genes (see STAR Methods) in BMDMs in the indicated experimental conditions. Data from two biological replicates. Pearson correlation > 0.97 for all replicates. Numbers indicate p-values (Mann-Whitney U test) for the corresponding comparisons.

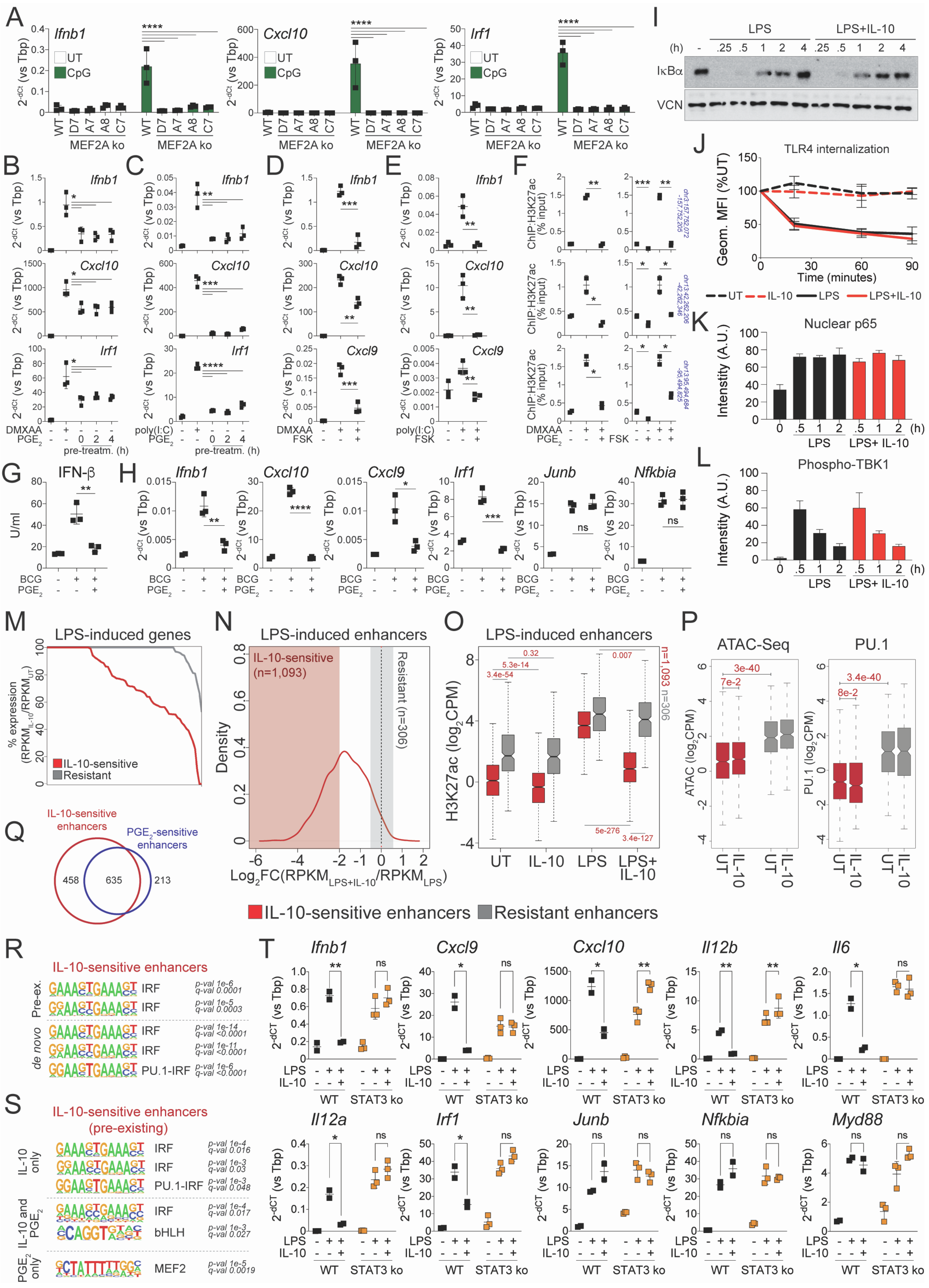

A
Ifnb1
Cxcl10
Irf1
UT
CpG
2-dCt (vs Tbp)
WT D7 A7 A8 C7
MEF2A ko
I
LPS
LPS+IL-10
(h)
IκBα
VCN
J
TLR4 internalization
Geom. MFI (%UT)
Time (minutes)
UT
IL-10
LPS
LPS+IL-10
B
C
D
E
F
Cxcl9
ChIP:H3K27ac (% input)
DMXAA
PGE2
pre-treatm. (h)
poly(I:C)
FSK
K
Nuclear p65
Intensity (A.U.)
LPS+ IL-10
L
Phospho-TBK1
G
IFN-β
U/ml
BCG
H
Junb
Nfkbia
ns
M
LPS-induced genes
% expression (RPKMIL-10/RPKMUT)
IL-10-sensitive
Resistant
N
LPS-induced enhancers
IL-10-sensitive (n=1,093)
Resistant (n=306)
Density
Log2FC(RPKMLPS+IL-10/RPKMLPS)
O
LPS-induced enhancers
H3K27ac (log2CPM)
n=1,093
n=306
UT
IL-10
LPS
LPS+ IL-10
P
ATAC-Seq
PU.1
ATAC (log2CPM)
PU.1 (log2CPM)
Q
IL-10-sensitive enhancers
PGE2-sensitive enhancers
458
635
213
IL-10-sensitive enhancers
Resistant enhancers
R
IL-10-sensitive enhancers
Pre-ex.
de novo
IRF
PU.1-IRF
S
IL-10-sensitive enhancers (pre-existing)
IL-10 only
IL-10 and PGE2
PGE2 only
bHLH
MEF2
T
Il12b
Il6
Il12a
Myd88
2-dCT (vs Tbp)
LPS
IL-10
WT
STAT3 ko

Figure S7. Impact of PGE_2 and IL-10 on inflammatory gene expression and genomic properties of IL-10-sensitive enhancers, Related to Figure 7. (A) RT-qPCR analysis of *Ifnb1*, *Cxcl10,* or *Irf1* in wt or *Mef2a*$^{-/-}$ iMacs stimulated for 4 hours with CpG. Genotypes of the individual clones are shown. Bar plots represent mean ± SD. Data from three biological replicates. **** p<0.0001 (two-way ANOVA test). (B-C) RT-qPCR analysis of *Ifnb1*, *Cxcl10* or *Irf1* in BMDMs stimulated with DMXAA (B) or poly(I:C) (C) in the absence or presence of PGE_2. The duration of PGE_2 pre-treatment is shown. Dot plots represent mean ± SD. Data from three biological replicates. **** p<0.0001, *** p<0.001, ** p<0.01, * p<0.05 (unpaired *t*-test). (D-E) RT-qPCR analysis of *Ifnb1*, *Cxcl10* or *Cxcl9* in BMDMs stimulated with DMXAA (D) or poly(I:C) (E) in the absence or presence of forskolin (2-hours pre-treatment). Dot plots represent mean ± SD. Data from three biological replicates. *** p<0.001, ** p<0.01 (unpaired *t*-test). (F) ChIP-qPCR analysis of a set of PGE_2-sensitive enhancers in BMDMs treated for 4 hours with DMXAA in the absence or presence of PGE_2 (left) or forskolin (right). Dot plots represent mean ± SD. Data from two biological replicates. *** p<0.001, ** p<0.01, * p<0.05 (unpaired *t*-test). (G) IFN-β release by BMDMs infected for 24 hours with BCG in the absence or presence of PGE_2. Dot plot represents mean ± SD. Data from two biological replicates. ** p<0.01 (unpaired *t*-test). (H) RT-qPCR analysis of a set of PGE_2-sensitive or resistant genes in BMDMs infected with BCG in the absence or presence of PGE_2. Dot plots represent mean ± SD. Data from two biological replicates. **** p<0.0001, *** p<0.001, ** p<0.01, * p<0.05, ns not significant (unpaired *t*-test). (I) Western blot analyses of IκBα in whole cell extracts in BMDMs stimulated with LPS or LPS+IL-10 for the indicated time points. (J) Flow cytometry analysis of TLR4 internalization showing percentage of geometric Mean Fluorescence Intensity relative to the untreated condition in BMDMs stimulated with control, LPS, IL-10 or LPS+IL-10 for the indicated time points. Line plot represents mean ± SD. Data from three biological replicates. (K-L) Immunofluorescence analyses of nuclear NF-κB p65 (K) or phosphorylated TBK1 (L) in BMDMs stimulated as indicated. Bar plots represent mean ± SD. Data from three biological replicates. (M) Expression of IL-10-sensitive or resistant genes as percent ratio of BMDMs treated with IL-10 to untreated controls. Data from three biological replicates. Pearson correlation > 0.97 for all replicates. (N) Density plot showing the effect of IL-10 costimulation on LPS-induced H3K27ac. Dotted line indicates lack of effect of the costimulation; red or grey shaded areas indicate values used to define IL-10-sensitive or resistant enhancers, respectively. (O) H3K27ac ChIP-Seq mean signal intensity within IL-10-sensitive (red) or resistant enhancers (grey) in the indicated conditions. Data from three biological replicates. Pearson correlation > 0.94 for all replicates. Numbers indicate p-values (Mann-Whitney U test) for the indicated comparisons. (P) ATAC-Seq (left) and PU.1 ChIP-Seq (right) signal intensities within pre-existing and *de novo* OCRs in IL-10 sensitive (red) or resistant (grey) LPS-inducible enhancers. Numbers indicate p-values (Mann-Whitney U test) for the indicated comparisons. (Q) Venn diagram showing the overlap between PGE_2-sensitive (blue) and IL-10-sensitive (red) enhancers. (R-S) Motif enrichment analysis showing top-ranking motifs identified within pre-existing or *de novo* IL-10-sensitive enhancers (R) or within pre-existing sensitive enhancers targeted by IL-10 only, by both IL-10 and PGE_2, and by PGE_2 (see STAR Methods) (S). Putative cognate TF families and associated p-values and q-values are shown. (T) RT-qPCR analysis of IL-10-sensitive and resistant genes in wt (black) or

STAT3-deficient (orange) BMDMs stimulated as indicated. Dot plots represent mean $\pm$ SD. Data from two or three biological replicates. ** $p<0.01$, * $p<0.05$, ns not significant (unpaired *t*-test).
